# Optimal selection of daily satellite precipitation product based on structural similarity index at 1 km resolution for the Pra catchment, Ghana

**DOI:** 10.1038/s41598-023-43075-0

**Published:** 2023-10-04

**Authors:** Yeboah Gyasi-Agyei, Emmanuel Obuobie, Bofu Yu, Martin Addi, Bashiru Yahaya

**Affiliations:** 1 School of Engineering and Built Environment, Griffith University, Nathan, Australia; 2 Water Research Institute, Council for Scientific and Industrial Research, Accra, Ghana; 3 Meteorology and Climate Science Department, Kwame Nkrumah University of Science and Technology, Kumasi, Ghana; 4 Remote Sensing and Climate Centre, Ghana Space Science and Technology Institute, Ghana Atomic Energy Commission, Accra, Ghana; 5 Ghana Meteorological Agency, Accra, Ghana

**Keywords:** Climate sciences, Hydrology

## Abstract

Thirteen satellite precipitation products (SPPs), re-gridded to 1 km resolution, were evaluated in terms of the structural similarity index (SSI) over the Pra catchment in Ghana. Three SPP scenarios were considered: Scenario one (S1) was the original SPPs; Scenario two (S2) was bias-corrected SPPs; and Scenario three (S3) was the better of S1 and S2 for each wet day. For each scenario, the best SPP was selected to constitute the 14th SPP referred to as the BEST SPP. Each SPP was evaluated in terms of SSI against the rain gauge rainfield for each wet day. For S1, the top three SPPs were TMPA, GSMAP and CMORPH; for S2, CMORPH, PERCCS and MSWEP were the top three; and for S3, CMORPH, PERCCS and TMPA came out on top in order of decreasing performance. Bias correction led to improvement in the overall SSI measure (SSIM) for 73% of wet days. The BEST SPP increased the SSIM of the best individual SPP by over 50% for S1, and over 30% for both S2 and S3. Comparing the BEST SPP of the three scenarios, S2 increased the SSIM statistic by 20% over that for S1, and SSIM was further improved by 4% for S3. It is highly recommended to use BEST SPP (S3) to generate the required 1 km × 1 km rainfields for the Pra, or other catchments around the world with a sparse rain gauge network, through conditional merging with rain gauge data as demonstrated.

## Introduction

CREAM (Building **C**limate **Re**silience into Basin W**a**ter **M**anagement) Project seeks to increase river basin resilience against climate change challenges to water infrastructure, livelihood, water-food-energy security, and environmental conservation. One aspect of the project seeks to develop a 1 km × 1 km grid of daily rainfield time series over the Pra catchment in Ghana to serve as input to rainfall-runoff models to generate runoff time series for water resources assessment in the catchment. A rainfield is defined as an area a rain system covers. However, the rain gauge network density over the catchment is very sparse (~ 1 per 300 km^2^), calling for innovative methods to generate the required rainfields. The sparsity of rain gauges is a common problem worldwide, making modelling of complex rainfields with high spatial and temporal variabilities a major challenge^1,2^. Moreover, it is rare to find rain gauges with a complete record without missing values due to factors such as equipment malfunction and accessibility^3–5^.

Traditionally, point data collected with rain gauges are interpolated over a grid to cover a catchment of interest using methods such as multiple regression and geostatistics^6–8^. However, sparsity of rain gauges limits the ability of these methods to achieve desired results as the spatial correlation cannot be reliably preserved as the rain gauge density decreases^9,10^. Radar technology has been used to monitor rainfall to address the low density of gauge networks, but this approach is very costly, and many developing countries cannot afford it. In addition, radar technology suffers from problems such as radar beam blockade, abnormal propagation and missing rain clusters^11^. Satellite technology for monitoring rainfall is fast becoming available for all regions in the world. Compared with radar, the satellite sensors can cover much larger areas and handle a wide range of landscapes such as large lakes, deserts and high mountains, not to mention the vast expanse of oceans. While radar and satellite-based rainfall estimates have strong spatial coverage, they still require adjustments to local rain gauge data to be useful in practice. In this regard, several methods have been developed to integrate radar, satellite, atmospheric reanalysis data and observed rain gauge data^12–14^, termed multi-source rainfall merging.

Currently, there are several freely available satellite related precipitation products at different spatial and temporal resolutions. The sensor and retrieval algorithms used in satellite rainfall technology can be either visible infrared (VIS/IR), passive microwave (PMW), active microwave (AMW) and multi-sensor precipitation estimation (MPE)^15,16^. Also, some of the products involve atmospheric reanalysis data that combine numerical weather forecasts with rain gauge, radar and satellite datasets. Some of the products are ARC2 (Africa Rainfall Climatology Version 2)^17^, CHIRPS (Climate Hazards Group InfraRed Precipitation with Station data)^18^ and ERA5 (European ReAnalysis)^19^. Most of the satellite related precipitation products have been bias corrected with some rain gauge datasets, but they perform differently in different regions and thus call for region-specific evaluation and assessment.

Satellite precipitation products (SPPs) have been evaluated globally and at regional scales. Sun et al.^20^ evaluated several satellite related precipitation products at different spatio-temporal scales globally using Taylor diagrams. They found significant differences in terms of the magnitude and variability, and no single product could be identified as the best. This is largely due to the different sensors and rainfall retrieval algorithms, the complexity of the rainfall processes, spatial coverage of rain gauge networks and region-specific characteristics. At the daily timescale and 0.5°, Islam et al.^21^ compared five SPPs against a gauge-based gridded daily rainfall dataset over the whole of Australia and found IMERG (Integrated Multi-satellitE Retrievals for Global Precipitation Measurement (GPM)) to provide the best results overall. Their analysis was based on bias ratio, correlation coefficient and the structural similarity index (SSI). Zhang et al.^22^ evaluated ARC2 and MSWEP (Multi-Source Weighted-Ensemble Precipitation) against rain gauge data at the daily and 0.25° spatial scales for the Sahel region based on correlation and trend analysis of rainfall variables. The seasonal pattern of rainfall for individual grid cells were compared. In general, there was agreement between the seasonal patterns except for the dry spells and the number of small events. Atiah et al.^23^ evaluated eight satellite precipitation products, including ARC2 and CHIRPS, at the monthly scale with a spatial resolution of 0.5° against gridded rain gauge data over Ghana using correlation coefficient, efficiency, bias and the root-mean-squared error as performance indicators. Their analysis was based on four agro-ecological zones of the country, using the point-to-pixel approach. They found that the performance of the products depends on the scale and location, and CHIRPS was generally better than other satellite precipitation products. Five SPPs were evaluated by Logah et al.^24^ over the Black Volta Basin, West Africa, using point-to-grid analysis, and using bias, correlation, Nash–Sutcliffe efficiency, probability of detection and false alarm ratio. CHIRPS emerged as the best SPP from their study followed by PERSIANN-CDR (Precipitation Estimation from Remotely Sensed Information using Artificial Neural Networks—Climate Data Record). Also using point-to-grid approach and correlation and bias, Owusu et al.^25^ evaluated three SPPs over the Pra catchment in Ghana at 0.25° spatial scale and daily, monthly, seasonal, and annual timescales. Their analysis identified TMPA-3B42 (Tropical Rainfall Measuring Mission Multi-satellite Precipitation Analysis) as the best SPP, while CMORPH (Climate Prediction Center morphing technique) overestimated rainfall at all gauge locations.

While there have been several attempts to evaluate SPPs, most of these were undertaken at coarse temporal and spatial scales of the original SPPs. The objective of this paper was to develop 1 km × 1 km rainfields at the daily timescale and to assess the quality of the generated rainfields to meet hydrological model requirements for flood studies and water resources assessment over the Pra catchment in Ghana, West Africa. Freely available SPPs were re-gridded to the required spatial scale before evaluation against rain gauge data. In particular, we sought to select the best SPP product for each day to obtain a time series from different SPP sources based on SSI. The methodology involves bias correction of the SPPs and conditional merging with rain gauge data through ordinary kriging. Section “Study area and data” provides an overview of the study area and data used followed by the methodology in section “Methodology”. Section “Results and discussion” presents the results and discussion, with the concluding remarks in section “Conclusions”.

## Study area and data

### Study area

**Figure 1:**
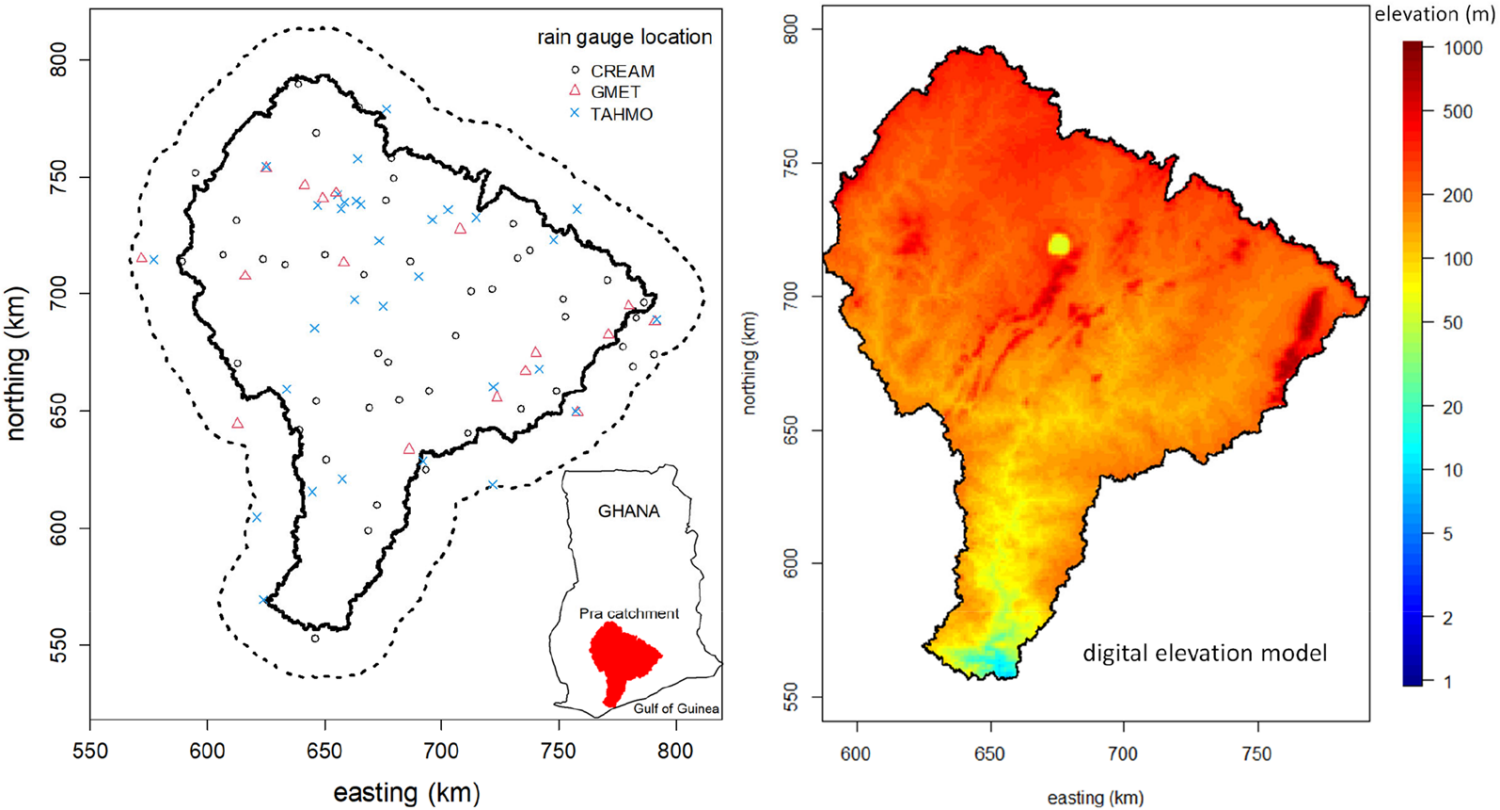
Left panel: location of the Pra catchment in Ghana (solid line) with 20 km buffer around the catchment (dashed line). The points are the rain gauge locations established by the various agencies. Right panel: digital elevation model of the Pra catchment. The maximum elevation is 870 m and the average is 200 m above mean sea level. The total catchment area is 23,262.7 km^2^, of which about 400 km^2^ drains into Lake Bosomtwe shown as a yellow circle in the middle of the catchment. Both maps were produced using R software version 4.2.1 (https://cran.rproject.org/).

In Fig. 1 (left panel) is shown the location of the Pra catchment, within Ghana, West Africa, and its digital elevation model is depicted in Fig. 1 (right panel). The catchment has a drainage area of 23,263 km^2^.The climate of the Pra catchment is classified as tropical monsoon with a dry and bimodal wet rainfall patterns that are controlled by the seasonal movement of the Inter-tropical Discontinuity (ITD)^26^. The ITD is the demarcation line that separates the north/north-eastern winds (hot, dry and dusty) from the Sahara and the south/south-western winds (cool and moist) from the Atlantic Ocean. Normally, the minor rainfall season occurs from September to October, and the major one from March to July. Annual rainfall over the catchment varies between 1000 and 1750 mm with a long-term average of 1200 mm. Generally, the average rainfall increases in the north-east to south-west direction, with the mean annual number of wet days between 90 and 100^27^. The relative humidity is between 50 and 60% during the dry season and between 70 and 80% during the wet season. The average annual temperature is about 28 °C, but above 30 °C is common in March and April, and the lowest of about 26 °C is registered in August.

### Daily rainfall gauge data

Daily rainfall data were sourced from: the CREAM Project (since 2019), TAHMO (**T**rans-**A**frican **H**ydro-**M**eteorological **O**bservatory) Project (since 2015, https://tahmo.org/) and **G**hana **Met**eorological Agency (GMet). GMet (https://www.meteo.gov.gh/gmet/) is responsible for setting up and monitoring the rain gauge network in Ghana and has long records of data for some stations dating back to 1960. Figure 1 (left panel) shows the 77 daily rain gauges located within the Pra catchment (GMet = 19, CREAM = 36, TAHMO = 22) and additional 21 (GMET = 3, CREAM = 9, TAHMO = 9) within the 20 km buffer around the catchment. Thus, the rain gauge density of the Pra catchment is about 1 per 300 km^2^ and the mean inter-station distance is about 17 km.

### The Satellite precipitation products (SPPs)

Data from 13 satellite precipitation products (SPPs) covering the Pra catchment and the 20 km buffer area were used in this study. Some of the SPPs are reanalysis datasets having satellite products components but, for simplicity, they are all referred to as SPPs. A brief description of each product is provided in the supplementary material Section 1 and their meta data are provided in Table 1.

**Table 1:** Meta data of the satellite precipitation products (SPPs) (all data used were up to 2020-12-31 except TMPA which ended on 2019-12-31 and W5E5V2 on 2020-01-01).

Satellite product (SPP)	Starting date	Temporal resolution	Spatial resolution (°)	Latency	IDW grid size (km)
ARC2	1984-01-01	00:00–23:59 GMT	0.1	~ 24 h	10
CHIRPS	1981-01-01	00:00–23:59GMT	0.05	~ 3 weeks	5
CMORPH	1998-01-01	0.5 h	0.0728	~ 18 h	7
ERA5	1981-01-01	1 h	0.1	~ 2 months	10
GSMAP	2001-01-03	1 h	0.1	~ 48 h	10
IMERG	2003-01-01	0.5 h	0.1	~ 3 months	10
MSWEP	1979-01-01	3 h	0.1	~ 3 months	10
PERCCS	2003-01-01	3 h	0.04	~ 1 h	4
PERDIR	2000-03-01	3 h	0.04	~ 1 h	4
PERSIANN	2000-03-01	3 h	0.25	~ 48 h	25
TAMSAT	1983-01-12	12:00–11:59 GMT	0.0375	~ 2 weeks	4
TMPA	2000-03-01	3 h	0.25	–	25
W5E5V2	1979-01-01	00:00–23:59 GMT	0.5	–	50

ARC2, **A**frica **R**ainfall **C**limatology Version **2**); CHIRPS, **C**limate **H**azards Group **I**nfra**R**ed **P**recipitation with **S**tation data; CMORPH, **C**limate Prediction Center **MORPH**ing; ERA5: **E**uropean **R**e**A**nalysis **5**th generation; GSMAP, **G**lobal **S**atellite **MA**pping of **P**recipitation; MSWEP, **M**ulti-**S**ource **W**eighted-**E**nsemble **P**recipitation; PERSIANN, **P**recipitation **E**stimation from **R**emotely **S**ensed **I**nformation using **A**rtificial **N**eural **N**etworks; PERCCS, **PER**SIANN-**C**loud **C**lassification **S**ystem; PERDIR, **PER**SIANN **D**ynamic **I**nfrared **R**ain; TAMSAT, **T**ropical **A**pplications of **M**eteorology using **SAT**ellite; TMPA, **T**ropical rainfall measuring mission **M**ulti-Satellite **P**recipitation **A**nalysis; W5E5V2, **W**ater and global change forcing data version **5** and **E**RA**5 V**ersion **2**; IDW, inverse distance weighting interpolation method.

#### Pre-processing of the SPPs

**Figure 2:**
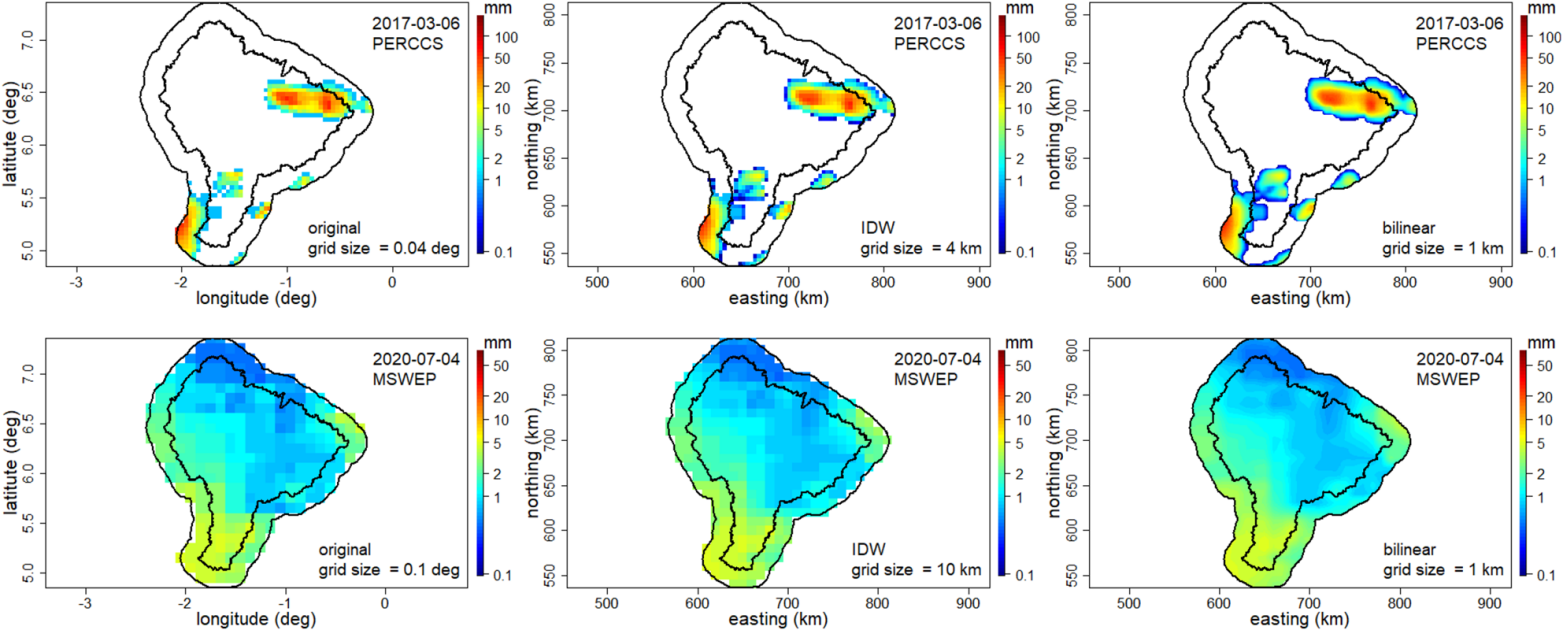
Comparison of the original rainfall (mm) satellite image in longitude-latitude coordinate system (left panels) with those after the initial interpolation using inverse distance weighting (IDW, middle panels) and the subsequent bilinear interpolation into 1 km × 1 km regular grid (right panels) in the easting-northing coordinate system. The top panels are for 2017-03-06 which is an example where only part of the catchment was wet as recorded by PERCSS and the bottom panels for 2020-07-04 is an example where the catchment experienced 100% wetness as recorded by MSWEP. The maps were produced using R software version 4.2.1 (https://cran.r-project.org/).

The SPPs were aggregated to the daily timescale to conform to the same temporal resolution as the daily rain gauge data, i.e., from 9 am of the previous day to 9 am of the present day. This temporal aggregation was not applied to ARC2, CHIRPS, TAMSAT and W5E5V2 as they were on a daily timescale, noting that there is a discrepancy of 3 h for TAMSAT and 9 h for ARC2, CHIRPS and W5E5V2. However, because the study area normally experiences short duration afternoon thunderstorms, the errors caused by the timings is not considered to be significant. First, the latitude–longitude coordinates of the SPPs were converted into the easting-northing coordinate system. This was followed by interpolation of the data into regular grids in km, the grid size of which varied (Table 1) depending on the original latitude–longitude grid size in degrees using the inverse distance weighting (IDW) method with 4 nearest neighbours. For example, the ARC2 dataset was converted to a regular grid of 10 km × 10 km because its spatial resolution was 0.10° (~ 10 km at the equator) and the CHIRPS to 5 km × 5 km because its spatial resolution was 0.05° (~ 5 km at the equator). All the SPPs were further re-gridded into a common 1 km × 1 km regular grid, and each grid cell considered to be small enough to assign a rain gauge to, using bilinear interpolation^28^. All the SPPs’ 1 km × 1 km regular grid conforms to the same grid centres. Two examples of the pre-processing of the original satellite products are shown in Fig. 2 for PERCSS on 2017-03-06 and MSWEP on 2020-07-04. As expected of any re-gridding technique, smoothing is achieved at the highest spatial resolution, but the spatial structure is very much preserved. These two examples were selected to show different catchment wetness.

The rain gauges were assigned to the closest grid cell, averaging multiple rain gauges if they were all in the same grid cell. In fact, only two grid cells, one at Kumasi Airport (x = 655.5 km, y = 742.5 km) and the other at Asamankese (x = 757.5 km, y = 649.5 km), contain two rain gauges, one each installed by GMET and TAHMO. While for some days the pairs of rain gauges recorded similar values, there were discrepancies observed for some days that could raise quality concerns for the average method. This issue will be investigated in further studies. Collocated datasets were extracted from the SPPs for the 1 km × 1 km grid cell where the rain gauge was located. This means that, for each day, the collocated dataset from the SPPs has the same coordinates and sample size as the rain gauge dataset. The rainfields of the rain gauge and the collocated data were developed by assigning missing values (NA as interpreted by the R programming language) for the grid centres without a rain gauge. As with the rain gauge dataset, there were days when SPPs’ data were missing.

## Methodology

The methodology builds on previous work on spatial modelling involving rain gauge, radar and satellite rainfall analysis^1,28–32^. It involves the following steps:

- identify the “true” cumulative distribution function (CDF) of the wet day rainfall pattern using the gauge, collocated and SPPs data;
- select the best SPP for each wet day through the SSI concept^33,34^;
- use the CDF to convert the rainfall amounts into Gaussian quantiles, and develop anisotropic correlograms from the SSP data;
- conditionally merge the Gaussianised quantiles of the SPP and rain gauge through ordinary kriging that uses the anisotropic correlograms;
- back transform the merged Gaussian rainfield to the ‘real’ rainfield at the 1 km × 1 km spatial scale.

Each aspect of the methodology is described in the following sub-sections.

### Data used

Unless otherwise stated, rainfall records used for the analysis were from 01/01/2017 to 31/12/2020. The number of operational rain gauges for the day varied considerably, largely due to the record length of the different data sources and faulty equipment. In total, 373 wet days that met the following criteria within the catchment and its 20 km buffer region were selected for analysis: (a) a minimum of 15 operational rain gauges for the day; (b) a minimum of 10 gauges with at least 0.1 mm of rain; (c) the average rainfall of the operational wet rain gauges is at least 1 mm; and d) data are available for all the 13 SPPs for an unbiased assessment. The period (4 years) of rainfall records used is not long, but the sample size of 373 wet days is adequate for SPP selection. In addition, the spatial pattern of the selected rainfall days is quite representative of the rainfall pattern in terms of its frequency and occurrence for the catchment.

### Identification of the “true” cumulative distribution function (CDF)

For each day, there are 13 SPPs, 13 collocated and 1 rain gauge datasets, making a total of 27 daily datasets from different sources. For each of the 27 daily datasets, the daily rainfall amounts greater than or equal to 0.1 mm (minimum threshold) were selected for fitting a two-parameter right-skewed distribution. The best distribution from a group of six, typically used for daily rainfall (Generalized Pareto, Gamma, Gumbel, Log-Logistic, Log-Normal, Kappa, and Weibull), was selected based on the Anderson–Darling statistic. The selected distribution, $F_{R}$, is then zero-inflated, $F_{R0}$, to accommodate the dry locations (grid cells) given as:

1 $$F_{R0} \left[ {s_{k} } \right]\quad = \quad \left\langle {\begin{array}{ll} {F_{R} \left( {r\left[ {s_{k} } \right]} \right)\left( {1 - p_{0} } \right) + p_{0} \quad ,{\kern 1pt} \quad r\left[ {s_{k} } \right] \ge 0.1\quad } \\ {p_{0} .\exp \left( { - \frac{{d\left[ {s_{k} } \right]}}{{\overline{d} }}} \right)\quad \quad \quad ,{\kern 1pt} \quad r\left[ {s_{k} } \right] < 0.1} \\ \end{array} } \right.$$

In Eq. (1) *k* is a grid cell number that varies from one to the maximum number of grid cells for the SPPs or rain gauges, *S*_*k*_ is the coordinates of grid cell *k*, $r\left[ {s_{k} } \right]$ is the rainfall amount at *S*_*k*_, *p*_*o*_ is the ratio of the number of dry grid cells (registering rainfall amounts < 0.1 mm) to the total number of grid cells, *d*[*s*_*k*_] is the distance of a dry grid cell from the nearest wet grid cell, and $\overline{d}$ is the average of *d*[*s*_*k*_] for all dry grid cells.

As a result of the small sample size of the rain gauge locations, and thus the collocated ones, the cumulative distribution of the SPPs and the rain gauge records could vary significantly^1^. An assumption of the traditional Quantile–Quantile (Q–Q) bias correction is that the rain gauge cumulative distribution function (CDF) is the correct one, and the satellite, radar or GCMs/RCMs CDFs need to be adjusted to suit^35^. Given the small sample size of the rain gauges, they may not provide a representative CDF, and this also applies to the rainfall spatial structure for that matter. Hence the bias correction procedure presented in Gyasi-Agyei^1^ is adopted here for estimating the “true” CDF (*F*_*TRUE*_), which is expressed as:

2 $$R(p)\quad = \quad F_{{_{TRUE} }}^{ - 1} (p)\quad = \quad F_{G0}^{ - 1} (p)\; + \;F_{S0}^{ - 1} (p)\; - \;F_{C0}^{ - 1} (p)$$

where *F*_*G0*_*, F*_*S0*_ and *F*_*C0*_ are zero-inflated fitted distributions of Eq. (1) for the rain gauge, each SPP and the collocated datasets for a given probability *p*, *F*^*−1*^ is the inverse of the CDFs and *R(p)* is the rainfall amount given by the “true” CDF for the given *p*. In essence, the rainfall amounts (*R*_*G*_) of the rain gauges are preserved but their probabilities (*p*_G0_) are corrected as:

3 $$p_{G0} \quad = \quad F_{TRUE} (R_{G} )$$

and, for the SPPs, the probabilities (*p*_*s*_) are preserved but the amounts (*R*_*S0*_) are corrected as:

4 $$R_{S0} \quad = \quad F_{TRUE}^{ - 1} \left[ {p_{S} } \right]$$

**Figure 3:**
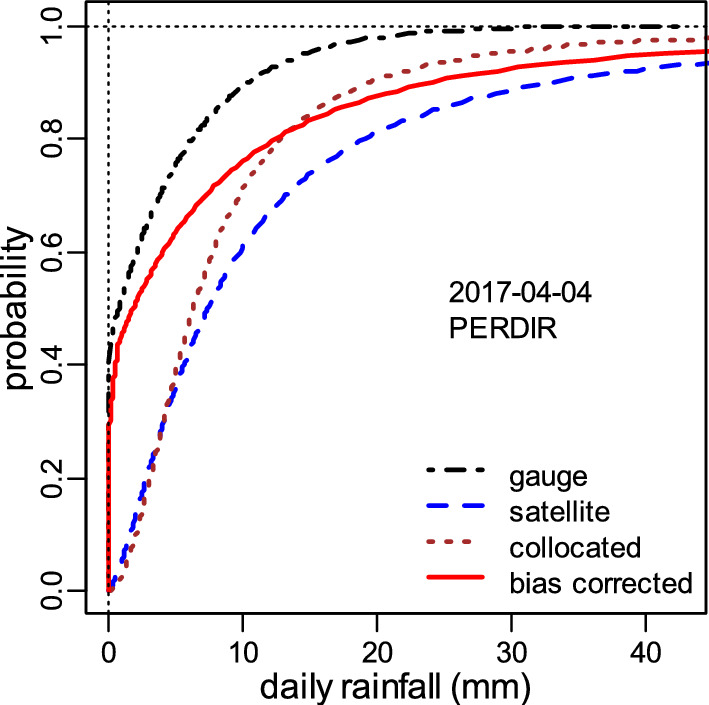
Identification of the true (bias corrected) cumulative distribution function (CDF) from the fitted zero-inflated CDF fitted to the rain gauge, PERDIR, and collocated datasets for 2017-04-04. For this example, the collocated CDF is drier than the full SSP, so it had to be stretched to the right to match the SSP. The same amount of stretch is applied to the CDF of the rain gauge.

For the example shown in Fig. 3, the collocated CDF is drier than the full SPP, so it had to be stretched to the right to match the SPP. The same amount of stretch is applied to the CDF of the rain gauge to obtain the “true” distribution.

### Selection of the best SPP for a wet day

The selection of the best SPP was based on the SSI methodology originally developed by Wang et al.^34^ for assessing image compression and enhanced by Jones et al.^33^. Islam et al.^21^ used the SSI methodology to evaluate five daily rainfall SPPs providing coverage of Australia at a grid scale of 0.5° (~ 50 km). It has also been used by Gyasi-Agyei^28^ to compare the performance of radar against three SPPs at the daily timescale within a weather radar station range in southeast Queensland, Australia. Here, it is used to compare the SPPs and the rain gauge rainfields.

**Figure 4:**
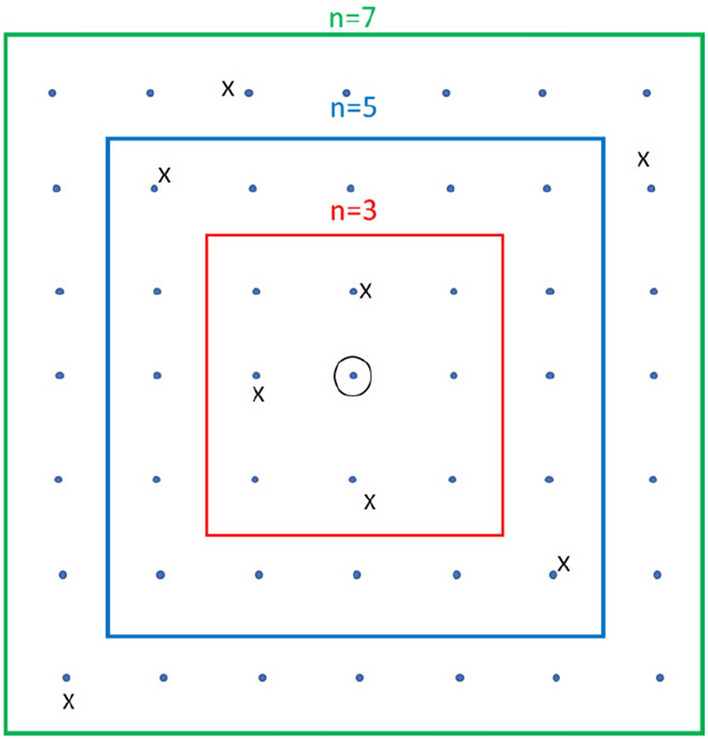
Windows over the circled grid centre; the inner window is of size 3 × 3, middle window of size 5 × 5, and the outer window of size 7 × 7 grid cells; the rain gauge locations are shown as ’x’.

With SSI as a quality indicator, a local window of spatial extent of *n* cells by *n* cells is moved over all the grid centres, as shown in Fig. 4, for *n* taking on values of 3, 5 and 7. Note that *n* should be an odd integer with a minimum of 3. Within each window, independent statistics of the local mean ($\mu_{k}$) and variance ($\sigma^{2}_{k}$) are calculated for the values of the SPPs and also for the rain gauge for cell *k* at the centre as^34^:

5 $$\mu_{k} \quad = \quad \sum\limits_{i = 1}^{{n^{2} }} {m_{i} } r_{i}$$

6 $$\sigma^{2}_{k} \quad = \quad \sum\limits_{i = 1}^{{n^{2} }} {m_{i} } \left( {r_{i} - \mu_{k} } \right)^{2}$$

where *r*_*i*_ is the rainfall amount for cell *i* having weight *m*_*i*_, the weights being considered as equal with a sum of unity, and *k* varies from 1 to the number of grid cells for the catchment. Next, the covariance ($C_{k}$) between the SPPs and the rain gauge rainfields is calculated as:

7 $$C_{k} \quad = \quad \sum\limits_{i = 1}^{{n^{2} }} {m_{i} } \left( {r_{i,S} - \mu_{k,S} } \right)\left( {r_{i,G} - \mu_{k,G} } \right)$$

where subscripts *S* and *G* refer to the parameters of the SPPs and rain gauge rainfields, respectively. These three statistics were used to calculate the spatial mean similarity (SIM_k_), variance similarity (SIV_k_), pattern similarity (SIP_k_) and the overall SSI measure of similarity (SSIM_k_) as:

8 $$SIM_{k} \quad = \quad \frac{{2\mu_{k,S} \mu_{k,G} + c_{1} }}{{\mu_{k,S}^{2} + \mu_{k,G}^{2} + c_{1} }}$$

9 $$SIV_{k} \quad = \quad \frac{{2\sigma_{k,S} \sigma_{k,G} + c_{2} }}{{\sigma_{k,S}^{2} + \sigma_{k,G}^{2} + c_{2} }}$$

10 $$SIP_{k} \quad = \quad \frac{{C_{k} + c_{3} }}{{\sigma_{k,S} \sigma_{k,G} + c_{3} }}$$

11 $$SSIM_{k} \quad = \quad SIM_{k} \, \cdot \,SIV_{k} \, \cdot \;SIP_{k}$$

In Eqs. (8–11), the constants are given as c_1_ = (0.01Q)^2^, c_2_ = (0.03Q)^2^ and c_3_ = c_2_/2 where Q is the range (difference between the maximum and minimum) of the values of the rainfall amounts of the SPPs and rain gauge rainfields being compared^34^. The Jones et al.^33^ reflection algorithm to correct edge effects by generating artificial buffers around the study area (including the 20 km buffer) was implemented, with the values of the reflected grid cells computed from the neighbouring grids. Hereafter, SIM, SIV, SIP and SSIM represent the mean of SIM_k_, SIV_k_, SIP_k_ and SSIM_k_, respectively, over all the 1 km × 1 km grid cells within the Pra catchment boundary only. Note that grid cells close to the buffer boundary do not have enough surrounding grid cells for the computation of the statistics and termed as edge effects.

All the statistics have a maximum value of 1 to signify complete similarity, and a minimum value of 0 for complete dissimilarity for both SIM and SIV while, for both SIP and SSIM, the complete dissimilarity value is − 1. Hence these statistics are valuable for assessing similarities between two rainfields that are difficult to discern by visual inspection. An implication for low similarity is that the high and low values do occur in different areas, while, for high similarity, they occur around the same area. The SSI statistics were evaluated for *n* taking on odd integer values between 3 and 11 inclusive, the upper limit being just above a spatial scale of 10 km. Grid cells without values for the rain gauge and collocated datasets were assigned NA (“Not Available”, the interpretation of missing values in R programming language). Increasing the window size smooths out the fine scale differences. Gyasi-Agyei^28^ recommended SSI indices above 0.75 as excellent, above 0.5 as good, and above 0.25 as satisfactory. For the ensuing analysis, only values of the largest window of 11 km by 11 km were used.

Three SPP dataset scenarios were considered: Scenario 1 (S1)—rainfields of the original SPPs; Scenario 2 (S2)—the bias-corrected SPPs using the rain gauge data; and Scenario 3 (S3)—the better (higher SSIM value) of S1 and S2 for each day. For each scenario, the best SPP for each day was selected to constitute the 14^th^ SPP referred to as the BEST SPP.

### Anisotropic correlogram

The anisotropic correlogram is the spatial structure required for ordinary kriging, and it is determined in the Gaussian (normal) quantile domain as required by the normality assumption of kriging. For each of the SPPs, Eq. (1) was used to estimate the Gaussianised rainfields (*w*) as:

12 $$w\left[ {s_{k} } \right]\quad = \quad \Phi^{ - 1} \left( {F_{R0} \left[ {s_{k} } \right]} \right)$$

where $\Phi^{ - 1}$ is the inverse of the normal quantiles $\Phi$. Note that the inversion of Eq. (12) to obtain rainfall amounts from the normal quantiles is represented as:

13 $$r\left[ {s_{k} } \right]\quad = \quad \left\langle {\begin{array}{*{20}c} {F_{R0}^{ - 1} \left[ {\left\{ {\Phi \left( {w\left[ {s_{k} } \right]} \right) - p_{0} } \right\}/\left( {1 - p_{0} } \right)} \right]\quad ,{\kern 1pt} \quad \Phi \left( {w\left[ {s_{k} } \right]} \right) \ge p_{0} } \\ {\quad \quad 0\quad \quad \quad \quad {\kern 1pt} \quad \quad \quad \quad \quad \quad \quad ,{\kern 1pt} \quad \Phi \left( {w\left[ {s_{k} } \right]} \right) < p_{0} } \\ \end{array} } \right.$$

**Figure 5:**
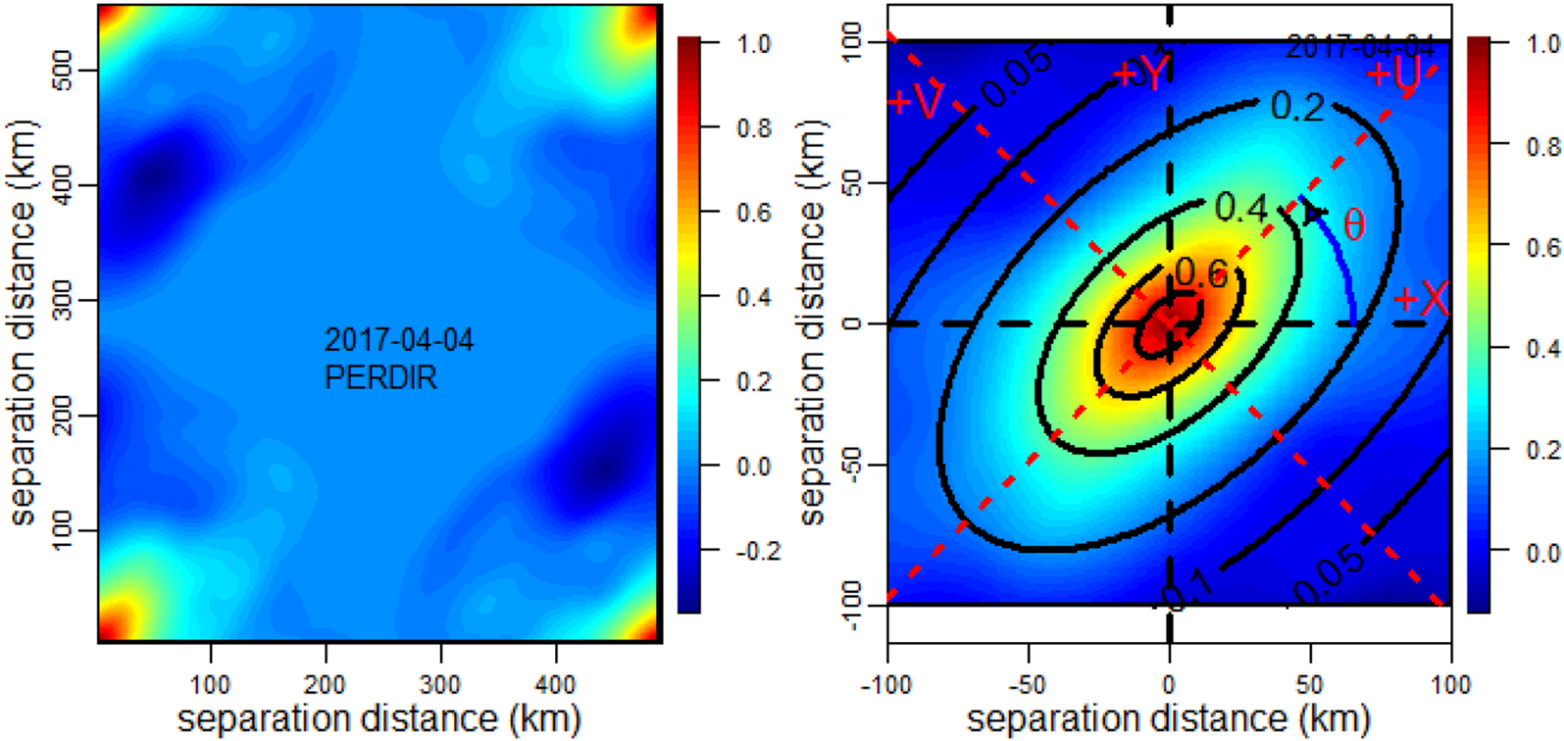
Left panel—correlogram before folding the four corners; Right panel—the final correlogram after folding the four corners for the central 200 km × 200 km. The fitted anisotropy exponential model parameters were *Lu* = 62 km, *Lv* = 34 km and *θ*  = 44°, and the elliptical contours are for the correlation set of {0.05, 0.1, 0.2, 0.4, 0.6 and 0.8}. This is for PERDIR on 2017-04-04.

There are two methods for estimating the sample correlogram of the Gaussianised rainfield, namely the classical moments approach and the Fast Fourier Transform (FFT) approach based on the power spectrum. Where the number of data points is limited, such as rain gauge locations, the classical moments approach is preferred as the FFT approach is not suitable. In the case of the SPPs where a large number of data points are under consideration, the FFT approach is superior and much faster in terms of computation time^29^, and has previously been used to model anisotropic correlograms^36^. The steps involved in the FFT approach are as follows:

- zero-inflate the Gaussianised rainfield to two times the size along each axis;
- transform the space domain to the frequency domain using FFT of the zero-inflated rainfield;
- take the square of the modulus of the FFT to obtain the power spectrum;
- the correlogram is obtained as the inverse FFT of the power spectrum in accordance with the Wiener–Khinchin Theorem;
- scale the correlogram such that the maximum occurring at the corners is 1 as per the definition of correlations (Fig. 5, left panel);
- fold the four quarters of correlogram to the centre, i.e., rotate each quadrant through 180°;
- select the central square of 200 km × 200 km to represent the sample correlogram as beyond this domain the correlation is negligible (Fig. 5, right panel).

The sample correlogram is fitted with a two-dimensional (2D) exponential model expressed as:

14 $$R_{\Theta } \left( {x,y} \right) = R_{\Theta } \left( {u,v} \right)\quad = \quad \exp \left\{ { - \left[ {\left( {\frac{u}{{L_{u} }}} \right)^{2} + \left( {\frac{v}{{L_{v} }}} \right)^{2} } \right]^{1/2} } \right\}\quad ,{\kern 1pt} \quad \begin{array}{*{20}c} {u = y\sin (\theta ) + x\cos (\theta )} \\ {v = y\cos (\theta ) - x\sin (\theta )} \\ \end{array}$$

where *x* and* y* are the separation distances in the cartesian coordinates (X, Y), and *v* and *u* are separation distances in the direction of the minor (V) and major (U) axes along an elliptical contour as defined in Fig. 5 right panel. The anisotropic ratio is defined as *η* = *L*_*v*_/*L*_*u*_ where *L*_*v*_ and *L*_*u*_ are the minor and major axis lengths, respectively, and *θ* is the anisotropic angle measured anticlockwise from the + X horizontal direction to the major axis. Hence the parameter set (*L*_*v*_, *L*_*u*_*, θ*) defines the 2D anisotropic exponential model which was optimised to match the sample and the analytical elliptical correlogram contours. Figure 5 shows the notations defining Eq. (14) for *L*_*u*_ = 62 km, *L*_*v*_ = 34 km and *θ *= 44°, and the elliptical contours for the correlation set of (0.05, 0.1, 0.2, 0.4, 0.6 and 0.8), as calculated for PERDIR on 2017-04-04.

### Conditional merging of the SPPs with the rain gauge data using Ordinary Kriging

Gyasi-Agyei and Pegram^29^ used the conditional merging method proposed by Sinclair and Pegram^37^ for simulated Gaussian rainfields and rain gauge data. Here, we replace the simulated Gaussian rainfields with the SPPs, and the correlogram required for kriging is obtained by the FFT on the Gaussian quantiles of the SPPs as described in section “Anisotropic correlogram”. In summary, the conditional merging approach is as follows:

- convert the rain gauge and the SPPs rainfall amounts into Gaussian quantiles as described in sections “Identification of the “true” cumulative distribution function (CDF)” and “Anisotropic correlogram”;
- interpolate the Gaussianised rain gauge data over the grid centres of the SPPs by ordinary kriging using the correlogram of the Gaussianised SPPs;
- the collocated data is extracted from each Gaussianised SPP and also interpolated as done for the Gaussianised rain gauge data using the same correlogram;
- the error rainfield is the difference between the Gaussianised SPPs and the interpolated collocated data at the grid centres;
- the error rainfield is added to the interpolated Gaussianised rain gauge data to obtain the conditionally merged rainfield in the Gaussian domain at each grid centre;
- use the Q–Q transform to convert the conditionally merged Gaussianised rainfield to the real rainfield in mm for the wet days, noting that the observed rain gauge amounts and the spatial structure of the SPPs are preserved.

## Results and discussion

### Structural similarity index (SSI)

For each of the 373 wet days, the SSI indices were calculated for the SPPs (including the BEST) and the gauge-based rainfield (taken as the “ground truth”) pairs over the selected 11 km × 11 km window size. Also, the analysis was done for both scenarios S1 and S2. The SPP registering the highest value of SSIM was selected as the best for the wet day. Where there was a tie, the first SPP was selected to get the same results each time the code was run. This occurred only once for S2 on 2019-03-15 where both CMORPH and PERCCS registered an SSIM value of 0.349. This means that the best SPP may be different for different days, and they constitute an additional set of SPPs referred to hereafter as BEST. Table 2 shows the proportion of days that each SPP emerged as the best, noting that prior to (S1) and post (S2) bias correction scenarios were conducted separately. The bias correction method may not work for some days due to the relative positions of the CDF of the rain gauge, collocated and SPP data. This is particularly true for cases where the CDF of the rain gauge is shifted too far to the left because of the CDF of the collocated data being below that of the SPP. Also, there could be fewer than three distinct wet day rainfall amounts for establishing the collocated CDF. For these reasons, it is germane to consider mixing the S1 and S2 scenarios in selecting the best SPP and not to rely on only one SPP. However, all the 373 wet days considered had good data for deriving S1 and S2 for all SPPs.

**Table 2:** Proportion of days each Satellite precipitation product (SPP) was the best for the 373 days.

No	Satellite precipitation product (SPP)	Prior bias correction Scenario 1, S1	Post bias correction Scenario 2, S2	Scenario 3, S3 best of S1 and S2
1	ARC2	8.0	4.8	6.2
2	CHIRPS	2.9	4.8	4.8
3	CMORPH	11.5	12.6	12.1
4	ERA5	2.4	11.3	10.5
5	GSMAP	22.8	11.8	11.5
6	IMERG	5.1	7.2	7.0
7	MSWEP	1.6	7.2	5.9
8	PERCCS	7.8	13.9	12.9
9	PERDIR	1.1	4.8	4.0
10	PERSIANN	6.4	5.1	4.6
11	TAMSAT	2.1	2.7	2.9
12	TMPA	12.6	4.8	5.4
13	W5E5V2	15.5	8.8	12.3
Standard deviation		6.45	3.64	3.60

Prior to bias correction, GSMAP had the highest proportion (22.8%) of the best SPPs, followed by W5E5V2 (15.5%) and then TMPA (12.6%). Post bias correction, the proportion of ERA5 increased significantly from 2.4 to 11.3% to be the fourth highest of the best SPPs, following PERCSS (13.2%), CMORPH (12.6%) and GSMAP (11.8%). From Table 2, notable decreases in the best proportions post bias correction were GSMAP (11%), TMPA (7.8%) and W5E5V2 (6.7%), because of the significant gains by ERA5 (8.9%), PERCSS (6.1%) and MSWEP (5.6%). The bias correction smoothed the variability among the 13 SPPs, with its standard deviation decreasing from 6.5 to 3.6% post bias correction. It needs to be underscored that, for some days, a few of the SPPs yielded similar SSIM values.

Prior to bias correction there were distinct groups of SPPs of similar SSIM values with the first group of CMORPH and GSMAP having values around 0.215, the second group of ARC2, PERSIAN and W5E5V2 having values of 0.195, the third group of IMERG and PERCSS having values of around 0.185, the fourth group of PERDIR and ERA5 having values of 0.153. MSWEP was the worst performing SPP (0.142) and TMPA the best (0.235) as shown in Table 3. These low SSIM values are a result of the low values of SIM prior to bias correction, and SIM improved noticeably with bias correction (Table 4). Table 5 shows further improvements in the SSI indices with Scenario 3. As shown in Table 6, the percentage improvement in the SSIM index with bias correction ranges from 20 to 114%; the highest improvement occurred for MSWEP followed by ERA5 (96%) The SIM index was the highest contributor to SSIM, with a range of 16% to 31%. For some SPPs, the SIP index decreased by up to 5% because of the bias correction. Thus, the role of bias correction is primarily to adjust the rainfall estimates to conform to those of the rain gauges. However, Scenario 3 further improved on Scenario 2, with SSIM values between 3 and 11% resulting from increases in SIP (varied between 1 and 8%) despite the insignificant reduction of less than 3% in SIV (Table 6).

**Table 3:** Average structural similarity indices (SSI) values for each SPP prior bias correction (Scenario 1, S1). SPPs are ranked in terms of SSIM values.

SPP	Rank	SSIM	SIM	SIV	SIP
BEST	1	0.353	0.518	0.719	0.783
ARC2	5	0.201	0.405	0.690	0.671
CHIRPS	10	0.167	0.395	0.664	0.629
CMORPH	4	0.215	0.394	0.705	0.680
ERA5	12	0.153	0.401	0.693	0.667
GSMAP	3	0.216	0.374	0.691	0.731
IMERG	8	0.188	0.402	0.689	0.639
MSWEP	14	0.142	0.399	0.695	0.641
PERCCS	9	0.183	0.380	0.693	0.640
PERDIR	11	0.153	0.383	0.686	0.609
PERSIANN	7	0.191	0.387	0.688	0.695
TAMSAT	13	0.148	0.404	0.684	0.616
TMPA	2	0.235	0.422	0.683	0.722
W5E5V2	6	0.195	0.413	0.684	0.745
Average (exclude BEST)		0.184	0.397	0.688	0.668
Stand. Dev. (exclude BEST)		0.029	0.014	0.009	0.044
Percent increase from Rank 2 to Rank 1		50.1	22.6	5.3	8.4

**Table 4:** Average structural similarity indices (SSI) values for each SPP post bias correction (Scenario 2, S2). SPPs are ranked in terms of SSIM values.

SPP	Rank	SSIM	SIM	SIV	SIP
BEST	1	0.424	0.603	0.797	0.752
ARC2	12	0.272	0.478	0.726	0.679
CHIRPS	14	0.257	0.486	0.712	0.638
CMORPH	2	0.312	0.486	0.738	0.721
ERA5	5	0.300	0.484	0.731	0.713
GSMAP	10	0.288	0.475	0.725	0.699
IMERG	6	0.292	0.489	0.735	0.683
MSWEP	4	0.303	0.498	0.741	0.700
PERCCS	3	0.309	0.500	0.740	0.694
PERDIR	8	0.289	0.483	0.730	0.687
PERSIANN	11	0.274	0.479	0.722	0.689
TAMSAT	13	0.269	0.485	0.722	0.659
TMPA	9	0.289	0.489	0.728	0.686
W5E5V2	7	0.292	0.489	0.721	0.722
Average (exclude BEST)		0.288	0.486	0.729	0.690
Stand. Dev. (exclude BEST)		0.016	0.007	0.009	0.023
Percent increase from Rank 2 to Rank 1		35.7	24.1	7.9	4.3

**Table 5:** Average structural similarity indices (SSI) values for each SPP—best of S1 and S2 scenarios (Scenario 3, S3). SPPs are ranked in terms of SSIM values.

SPP	Rank	SSIM	SIM	SIV	SIP
BEST	1	0.438	0.602	0.783	0.782
ARC2	12	0.297	0.485	0.719	0.713
CHIRPS	14	0.277	0.485	0.707	0.681
CMORPH	2	0.330	0.497	0.741	0.732
ERA5	6	0.318	0.506	0.719	0.740
GSMAP	8	0.315	0.478	0.721	0.745
IMERG	9	0.311	0.492	0.724	0.709
MSWEP	7	0.316	0.514	0.729	0.714
PERCCS	3	0.322	0.504	0.745	0.705
PERDIR	11	0.300	0.492	0.727	0.695
PERSIANN	10	0.301	0.488	0.711	0.727
TAMSAT	13	0.285	0.499	0.709	0.686
TMPA	4	0.321	0.495	0.707	0.742
W5E5V2	5	0.320	0.507	0.707	0.768
Average (exclude BEST)		0.309	0.496	0.720	0.720
Stand. Dev. (exclude BEST)		0.016	0.010	0.013	0.025
Percent increase from Rank 2 to Rank 1		32.9	21.0	5.6	7.0

**Table 6:** Percent change in the average structural similarity indices (SSI) values from Scenario 1 (Table 3) to Scenario 2 (Table 4) and from Scenario 2 (Table 4) to Scenario 3 (Table 5) for the individual SPPs and the BEST.

SPP	Percentage change in the indices from Scenario 1 to Scenario 2				Percentage change in the indices from Scenario 2 to Scenario 3			
	SSIM	SIM	SIV	SIP	SSIM	SIM	SIV	SIP
BEST	20.0	16.4	10.9	− 3.9	3.4	− 0.1	− 1.8	4.0
ARC2	35.9	18.0	5.3	1.1	9.0	1.5	− 1.0	5.0
CHIRPS	54.1	22.9	7.1	1.5	7.6	− 0.2	-0.7	6.7
CMORPH	45.2	23.2	4.8	6.0	5.5	2.4	0.3	1.5
ERA5	96.1	20.7	5.5	7.0	6.1	4.5	− 1.7	3.7
GSMap	33.4	27.0	4.9	− 4.3	9.2	0.7	− 0.6	6.5
IMERG	55.8	21.6	6.6	7.0	6.3	0.6	− 1.4	3.7
MSWEP	113.9	24.6	6.7	9.1	4.2	3.4	− 1.7	2.1
PERCCS	68.7	31.5	6.7	8.4	4.2	0.9	0.8	1.7
PERDIR	88.9	26.2	6.4	12.7	3.9	1.9	− 0.4	1.2
PERSIANN	43.1	23.8	5.0	− 0.9	9.8	1.9	− 1.6	5.6
TAMSAT	82.0	20.2	5.5	7.1	6.1	2.8	− 1.7	4.0
TMPA	22.8	15.8	6.6	− 5.0	11.1	1.1	− 2.8	8.2
W5E5V2	50.0	18.3	5.5	− 3.1	9.7	3.8	− 2.0	6.3

Prior to bias correction, the best 6 SPPs in decreasing values of SSIM index were TMPA, GSMAP, CMORPH, ARC2, W5E5V2 and PERSIANN (Table 3). Post bias correction, CMORPH emerged as the best SPP, closely followed by PERCCS, MSWEP, ERA5, IMERG and W5E5V2 (Table 4). For Scenario 3, which is much better than Scenarios 1 and 2, the ranking of the SPPs in decreasing order were CMORPH, PERCCS, TMPA, W5E5V2, ERA5, MSWEP, GSMAP, IMERG, PERSIANN, PERDIR, ARC2, TAMSA and CHIRPS (Table 5). It is worth noting that the differences in the SSI indices among the 13 SPPs for Scenario 3 were small, with mean values of 0.309 for SSIM, 0.498 for SIM, and 0.72 for both SIV and SIP.

The point-to-grid approach used by others (e.g.,^24,25^) is in fact a variant of our Scenario 1, with the grid size as a fixed window for calculating the SIM index. Owusu et al.^25^ found TMPA to be better than CMORPH for the Pra catchment, noting that they used data from 2003 to 2008 and only data from 7 rain gauges for the evaluation. This is supported by our Scenario 1 results where the SSIM value for CMORPH (0.215) was below that of TMPA (0.235). However, Scenario 2 (Table 4) involving bias correction improved all SPPs, but CMORPH (SSIM = 0.312) ranked better than TMPA (SSIM = 0.289). Over the Black Volta Basin which lies north of the Pra catchment, Logah et al.^24^ reported CHIRPS, PERSIANN, TMPA and ARC2 to be in a decreasing order of performance for rainfall, this being contrary to our rankings shown in Table 3 (Scenario 1) while TMPA emerged as the best among all the SPPs. Post bias correction though, CHIRPS was ranked at the bottom and TMPA at the top considering the set of SPPs they evaluated (Table 4). Their evaluation was based on 21 rainfall stations with daily data between 1981 and 2010. Evaluating five SPPs against a gauge-based gridded SILO dataset in Australia, Islam et al.^21^ observed that IMERG and TMPA outperformed CMORPH and PERSIANN. In comparison with our Scenario 1 results (Table 3), TMPA and CMORPH performed better than IMERG and PERSIANN.

Of particular interest is the performance of the BEST SPP, a conglomerate of the best of all SPPs for the individual wet days. It gained 22.6%, 5.3%, 8.4% and 50.1%, for SIM, SIV, SIP and SSIM statistics, respectively, compared with the best performing SPP of TMPA prior to the bias correction scenario (S1). Post bias correction (S2), the gains were 24.1%, 7.9%, 4.3% and 35.7% for SIM, SIV, SIP and SSIM, respectively, over the best SPP of CMORPH. For Scenario 3, the gains were, respectively, 21.1%, 5.7%, 6.8% and 32.8% for SIM, SIV, SIP and SSIM over the best SPP of CMORPH. Considering the BEST SPP of the Scenarios 1 and 2, the bias correction improved the SIM, SIV and SSIM statistics by 16.4%, 10.8% and 20.1%, respectively, but decreased the SIP value by 4%. Hence bias correction is most effective in improving the SIM statistic which in turn improves the overall SSIM. From Scenario 2 to Scenario 3, the improvement in SSIM was 3.3% largely contributed by SIP which increased by 4% with SIM and SIV showing minimal decrease of 0.16% and 1.75%, respectively.

**Figure 6:**
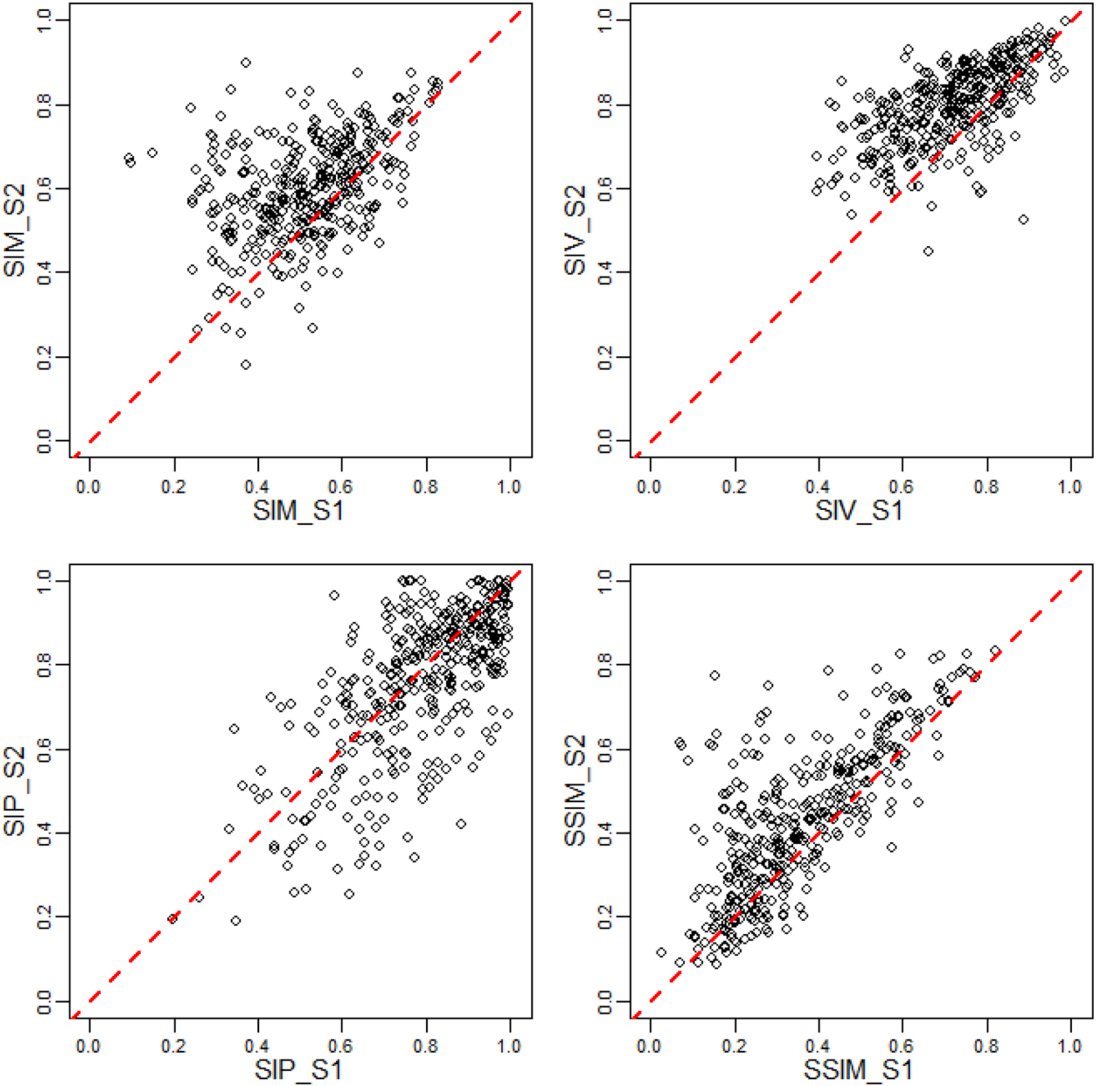
Improvement in terms of structural similarity indices (SSI) values for the best satellite precipitation product for each wet day due to bias correction. SSI values are for the window size of 11 km × 11 km. The dashed lines are for perfect agreement, i.e., points above the dashed lines indicate better performance of Scenario 2 (S2) over Scenario 1 (S1).

Out of the 373 wet days, only 72 (19.3%) have the same SPP as the best for both Scenarios 1 and 2, meaning the bias correction could cause changes in the preferred SPP for the wet day. For each wet day and scenario, the SSI statistics of Scenarios 1 and 2 are compared in Fig. 6. Points above the lines of perfect agreement indicate that bias correction has resulted in better performance. With bias correction, the percentage of wet days that the SSI statistics have improved were 72.9%, 79.6%, 42.1% and 72.9% for SIM, SIV, SIP and SSIM, respectively.

**Figure 7:**
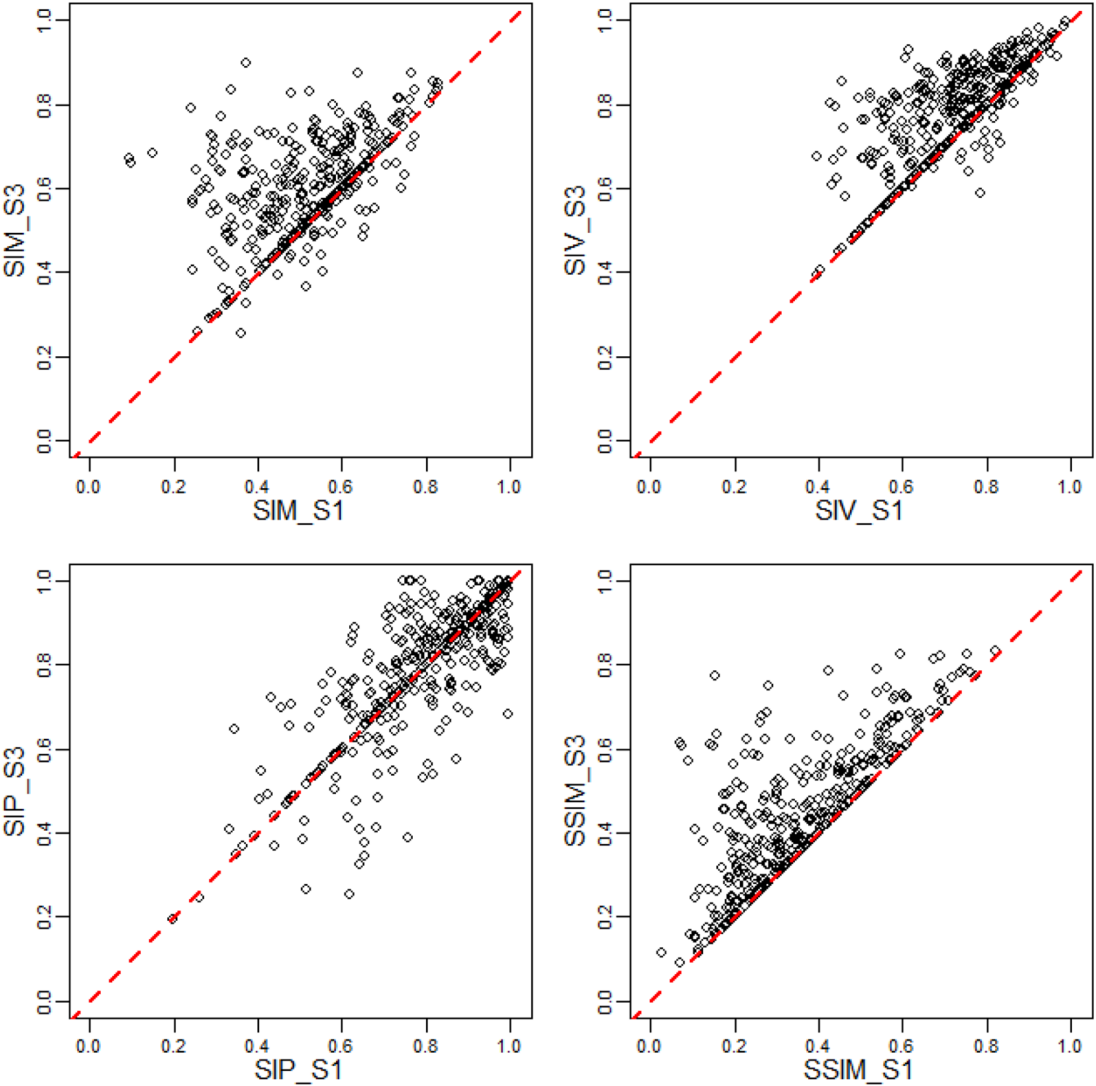
Improvement in terms of structural similarity indices (SSI) values for the best SPP for each wet day prior or post bias correction (Scenario 3, S3). SSI values for the largest window size (11 km × 11 km) were used. The dashed lines are for perfect agreement, i.e., points above the dashed lines indicate better performance of Scenario 3 (S3) over Scenario 1 (S1).

From Fig. 7, Scenario 3 resulted in improved SIM, SIV and SIP for 86.9%, 88.2% and 64.3% of wet days compared to Scenario 1. The composition of the best SPP of the three scenarios is presented in Table 2. ERA5 and PERCCS benefited the most in terms of the number of best SPPs resulting from the bias correction and amalgamation of Scenarios 1 and 2 at the expense of GSMAP, TMPA and W5E5V2.

**Figure 8:**
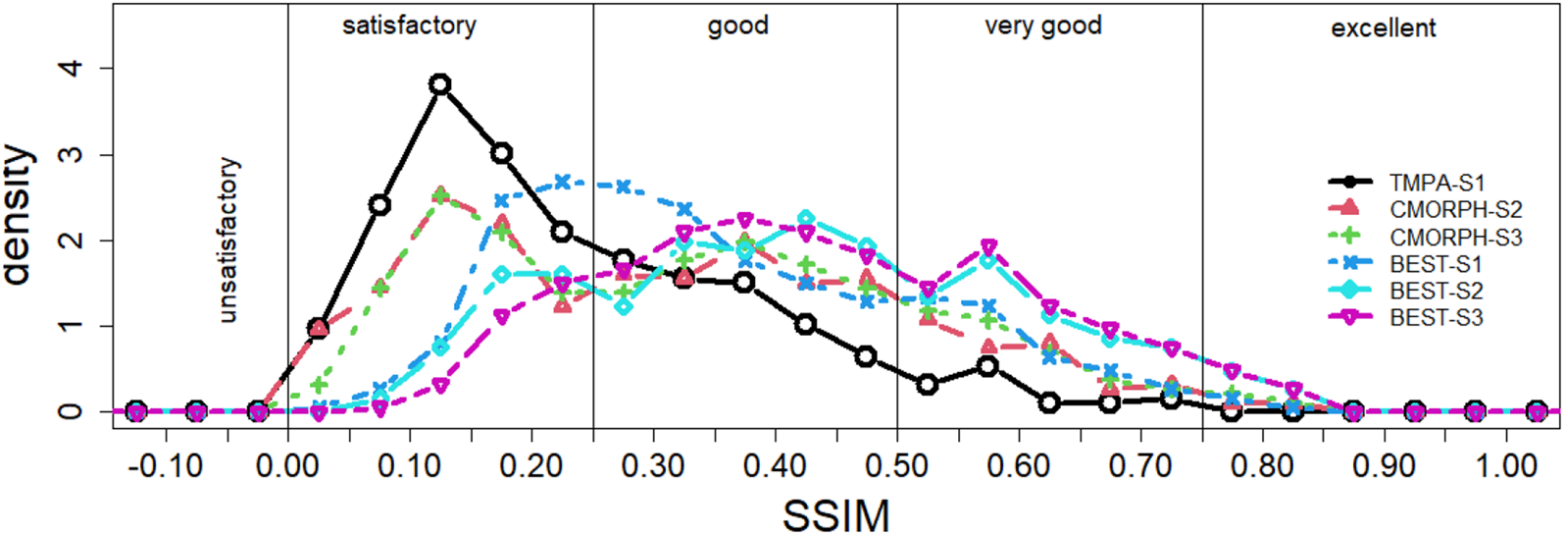
Density function of the overall measure of similarity (SSIM): TMPA is the best satellite precipitation product (SPP) for Scenario 1 (S1), CMORPH is the best SPP for Scenario 2 (S2), CMORPH is the best SPP for Scenario 3 (S3), BEST is the best SPP for each day for the three scenarios.

Figure 8 shows the density function of the SSIM values. Also shown in Fig. 8 is the recommended classification of the quality of the merged daily rainfields in terms of SSIM into unsatisfactory (SSIM ≤ 0), satisfactory (0 < SSIM ≤ 0.25), good (0.25 < SSIM ≤ 0.5), very good (0.5 < SSIM ≤ 0.75) and excellent (> 0.75). Note that SSIM is a product of three values, each having a maximum value of 1. Hence taking the cubic root of these limits could define the limits for SIM, SIV and SIP. The significant improvement post bias correction is demonstrated by the shift of the TMPA-S1 (best SPP for S1) curve to the CMORPH-S2 (the best SPP for S2) curve to the right for individual best SPPs. This is also demonstrated by the shift of the BEST-S1 curve to the BEST-S2 curve to the right. Further improvement in SSIM for Scenario 3 is depicted by the shift from the CMORPH-S2 curve to the CMORPH-S3 (best SPP for S3) curve to the right, and the marginal shift from the BEST-S2 curve to the BEST-S3 curve to the right. Based on the SSIM classification, the performance of all the 13 individual SPPs is considered good as they collectively registered SSIM > 0.28 for Scenario 3, but the BEST Scenario 3 is the preferred set of SPPs to be used to generate the required 1 km × 1 km rainfields for the Pra catchment.

For the IR retrieval algorithms, rainfall is estimated using statistical relationships between rainfall intensities and some properties of cloud field such as cloud top bright temperature detected by geostationary satellite sensors, and generally tend to under- or over- estimate rainfall but have good temporal scales^38^. Passive microwave retrieval algorithms based on polar-orbiting sensors are believed to provide better estimates because they can probe inside the cloud for rainfall information but have coarse spatial scales and have difficulties in regions of complex topography^39^. Therefore, SPPs that depend on IR and not on PMW (ARC2, CHIRPS, PERSIANN, PERCCS, PERDIR and TAMSAT) were expected to be outperformed by the others. However, bias correction of the SPPs with some ground data could alter this conclusion. A typical example is TMPA that emerged as the best SPP for Scenario 1 because it combines PMW and IR sensors and it has been bias corrected with ground data (Table 3). Surprisingly, MSWEP^12^ that merges several satellite products, gauge and reanalysis data ranked last for Scenario 1. ERA5 is also a composite of historical, satellite and ground radar data from several sources^19^ but ranked second from the bottom for Scenario 1. Evaluating SPPs over India, Prakash^40^ reported that MSWEP was outperformed by both CHIRPS and TMPA at the monthly timescale. However, bias correction (Scenario 2) with our data placed MSWEP in the third position (Table 4) and in the seventh position for Scenario 3 (Table 5). From a comprehensive review by Sun et al.^20^, it was observed that the different retrieval algorithms give significantly different rainfall output and no single SPP can be labelled as the best. It is also true from our analysis that the skills of the SPPs vary daily depending on the complex rainfall spatial structure of the day.

Analysis above shows that any of the SPPs can be improved through bias correction subject to the quality of the rain gauge data. Despite the growing list of SPPs freely available, it is necessary to validate the products with local rain gauge data before their usage. Hence the value of rain gauge data cannot be overstated, and the accuracy of the estimation of rainfields for hydrological modelling increases with increasing rain gauge network density^9,10^. Due to missing records, one cannot rely on just one SPP. Also, each of the products has its own strengths and weaknesses and may not be able to capture the complex nature of continuity and intermittency of rainfall that exhibits significant spatio-temporal variability daily. Thus, a combination of several SPPs as presented may be the way to optimise the use of the SPPs.

Given the mean inter-station distance of 17 km, some windows may not have a rain gauge to compute the SSI indices. This would have directly influenced the results. As seen in Fig. 1, some regions have denser rain gauge networks than others. Nevertheless, the SPPs were evaluated under the same conditions. For regions with a denser rain gauge network, a threshold on the minimum number of rain gauges per window could be set.

### Effects of the number of rain gauges on the structural similarity indices and rainfall estimates

**Figure 9:**
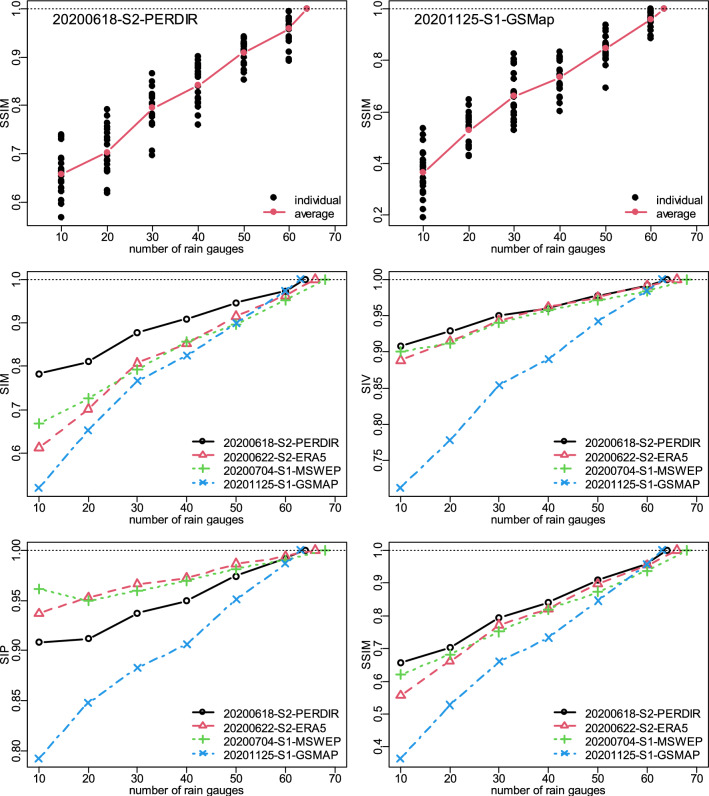
Effects of the number of rain gauges on the structural similarity indices comparing the merged rainfields of the highest number of rain gauges and the reduced numbers: S1 (Scenario 1) prior and S2 (Scenario 2) post bias correction. The top two panels show values of the individual repeated samples for a fixed number of gauges while the bottom four panels show the average of 20 repetitions for each number of rain gauges.

The number of rain gauges have a profound effect on the developed rainfields. An investigation was therefore carried out by varying the number of rain gauges by an increment of 10 starting from 10 to the maximum possible. The four days of 2020-06-18, 2020-06-22, 2020-07-04 and 2020-11-25 that had 64, 66, 68 and 63 operational rain gauges and different rainfield patterns and intensities (Supplementary material, Table S1) were analysed. For each of these days, analysis was carried out using their best SPPs for Scenarios 1 and 2 (Table S2). Given the number of rain gauges of interest as N (10, 20, 30, 40, 50, 60), N rain gauge locations were randomly sampled with 20 repetitions, from the operational rain gauge locations for the day, making sure at least 5 wet rain gauges were included. Each of the rain gauge sets together with the best SPPs were used to develop merged daily rainfields as discussed under the methodology. The structural similarity indices were calculated by comparing the generated conditional merged rainfields with the case of using the maximum number of operational rain gauges (considered the “truth”) to ascertain the loss of SSI indices due to reduction in the number of rain gauges. The top 2 panels of Fig. 9 show example results for the variability of the SSIM statistic of the 20 repeated samples for a given fixed number of rain gauges. They demonstrate the uncertainty of the SSI indices in relation to the location of gauges, noting that the rainfall pattern exhibits considerable daily variability over the catchment.

As demonstrated in the bottom four panels of Fig. 9, all the SPPs show a decline in the SSI indices as the number of rain gauges decreases. The SIM index exhibits the highest decline, varying from 0.0043 per gauge (PERDIR) to 0.009 per gauge (GSMAP) with a mean value of 0.0065 per gauge. With SIV, the rate of decline varied between 0.0017 per gauge (PERDIR, MSWEP) to 0.0054 per gauge (GSMAP) and had an average value of 0.0027 per gauge which was under half that of SIM. The smallest rate of decline occurred in SIP, having a mean value of 0.0019 per gauge, a minimum of 0.0007 per gauge (MSWEP) and a maximum of 0.004 per gauge (GSMAP). Hence the decline in SSIM, being a minimum of 0.0066 per gauge (MSWEP, PERDIR), a maximum of 0.012 per gauge (GSMAP), and an average of 0.0084 per gauge, can largely be attributed to the decline in SIM. For GSMAP, there is a sharp decline in SIM and SIP below 30 gauges which may signal inadequacy of less than 30 gauges for the analysis carried out in this paper. While the decline in the number of rain gauges up to 20 may not be much of a concern, having as many as 200 may help identify any break in scaling^41^ with the potential to address the optimum number of rain gauges for hydrological analysis that has gained research interest^1,2^. For fewer rain gauges, the key is where they are located on the catchment with respect to the rainfall pattern of the day which varies significantly as observed in the images presented in the Supplementary material Figures S1 to S7 introduced in section “Examples of the developed 1 km x 1 km daily rainfields”. However, it is worth noting that the SPPs evaluated were subjected to the same number and location of the rain gauges. Given the sparsity of rain gauge density of the Pra catchment, the analysis presented improves the use of SPPs for hydrological modelling and assessment. The focus of this paper was to reconstruct historical rainfields, so comparing SPPs of different latency (Table 1) was not a problem. For applications that require near-real-time data such as flood forecasting, SPPs of short latency of about one hour should be evaluated^28^.

**Figure 10:**
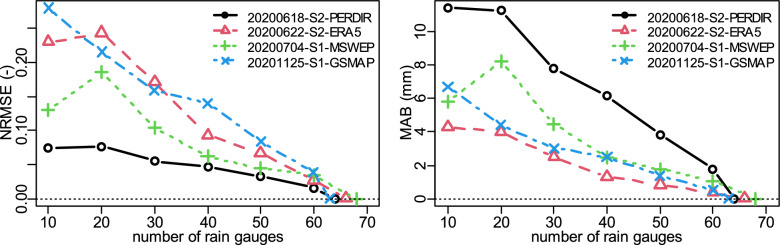
Effects of the number of rain gauges on the normalised root-mean-square-error (NRMSE) and mean bias (MAB) comparing the merged rainfields of the highest number of rain gauges and the reduced numbers: S1 (Scenario 1) prior and S2 (Scenario 2) post bias correction.

Uncertainty of the rainfall estimates because of the decreasing number of rain gauges was caried out using the conditionally merged rainfields with 20 repetitions and the performance statistics of normalised root-mean-square-error (NRMSE) and mean absolute bias (MAB) defined as:

15 $${\text{NRMSE}}\quad = \quad \sqrt {\frac{1}{N}\sum\limits_{k = 1}^{N} {\left[ {R_{O} (k)\; - \;R_{P} (k)} \right]^{2} } } /(R_{\max } - R_{\min } )$$

16 $${\text{MAB}}\quad = \quad \frac{1}{N}\sum\limits_{k = 1}^{N} {\left| {R_{O} (k)\; - \;R_{P} (k)} \right|}$$

where *R*_*O*_(*k*) and *R*_*P*_(*k*) are the values at grid cell *k* of the rainfield of maximum number of rain gauge and the other rainfields, respectively, *R*_*max*_ and *R*_*min*_ are the maximum and minimum values of *R*_*O*_ and *N* is the number of grid cells of the rainfield. Figure 10 shows that NRMSE and MAB increases with decreasing number of rain gauges. This trend is expected as the quality of the generated rainfield is reduced as the number of rain gauges decreases.

### Examples of the developed 1 km × 1 km daily rainfields

Seven wet days were selected to illustrate generated rainfields by conditional merging of the SPPs and rain gauge dataset of the individual days. Due to space consideration only seven, a number chosen arbitrarily, wet days of different rainfall pattern and characteristics, and of different number of rain gauges were presented. They were randomly selected from two groups, one having less than 30 rain gauges and the other over 60 rain gauges, to demonstrate the extreme ends of the number of rain gauges. Of particular interest was the investigation of the effect of the number of operational rain gauges on the SSI statistics. Table S1 shows a considerable variation of the number of wet gauges, the maximum gauge reading, the mean wet gauge reading, and the proportion of wet gauges of the seven selected wet days. The structural similarity indices for both prior (S1) and post (S2) bias correction are presented in Table S2 for the BEST SPP. There are days that the bias correction improved the SSIM through adjustment of the other structural similarity indices. Detailed discussion of the individual days, including the related tables and figures, are provided in the Supplementary materials.

## Conclusions

For many parts of the world, different sets of SPPs have been evaluated, many at the spatial scale of the original dataset, to identify the best for each catchment or region. Our approach was developed to generate daily time series of rainfields based on the best SPP among different SPPs. This approach has not previously been attempted nor evaluated based on what we can find in literature. In addition, daily SPPs (prior and post bias correction) were evaluated at 1 km scale, fine enough to assign a rain gauge to a grid cell to generate rainfields based on rain gauge. The SSI values were used to compare the SPPs and the rain gauge rainfields to select the best SPP for a wet day. Scenario 3 SPPs were constructed from the best out of Scenarios 1 (prior bias correction) and 2 (post bias correction), thus consisting of a mixture of the original and bias corrected SPPs. For each day, the best SPP was selected to constitute what we termed the “BEST” SPP. The selected SPPs were conditionally merged with the rain gauge data using ordinary kriging. Findings of the research are:

- No single SPP was consistently better than the other SPPs for all wet days. Only one fifth of the wet days were associated with the same SPP as the best for Scenarios 1 and 2, with Scenario 2 being better than Scenario 1 for about three quarters of the wet days.
- Bias correction (Scenario 2) significantly improved the SSI indices values for all SPPs, but not to the same level, and the level of improvement also varied among the SPPs. This led to the change in the preferred SPPs of Scenarios 1 and 2, and consequently that of Scenario 3. The bias correction improvement was more pronounced in the mean and the variance indices, but with barely any effect on the spatial pattern index.
- Without a doubt, mixing the SPPs, i.e., selecting the best SPP for each wet day either prior or post bias correction, is much superior to relying on any single SPP without bias correction.
- Record length and missing data are often problematic for a single SPP. Hence it is highly recommended to use Scenario 3 BEST SPPs to generate the required rainfields. Using the BEST SPPs resolves the potential issue of missing records and maximises the record length.
- SSI values increased markedly as the number of rain gauges in the catchment increased, implying a continuing need for ground-based precipitation observations.
- Under- and over-estimation of observed rainfall by the SPPs introduces uncertainties for their direct use. Conditional merging of the BEST SPPs with the rain gauge data improved the quality of the generated anisotropic rainfields over the original datasets.

Some aspects of the methodology have been applied in South Africa and Australia. Although not presented here, we have generated conditionally merged daily rainfields for water resources assessment from 1981 to 2020 for the Pra catchment. In fact, the methodology presented in this paper can be applied to any region in the world as satellite products are available globally.

### Supplementary Information

Supplementary Information.

## Data Availability

The datasets used and/or analysed during the current study are available from the corresponding author on reasonable request.

## Abbreviations

FFT: Fast Fourier Transform ()
ITD: Inter-tropical Discontinuity
S1: Scenario 1 consisting of rainfields of the original SPPs
S2: Scenario 2 consisting of the bias-corrected SPPs using the rain gauge data
S3: Scenario 3 is the better (higher SSIM value) of S1 and S2 for each day
Q–Q: Quantile–Quantile mapping
SIM: Spatial similarity in mean
SIP: Spatial similarity in pattern
SIV: Spatial similarity in variance
SSI: Structural similarity index
SSIM: Overall SSI measure of similarity

## List of symbols

$\mu_{k}$: Local mean of cells within a window centred on cell *k*
$\sigma^{2}_{k}$: Local variance of cells within a window centred on cell *k*
$\Phi$: Gaussian (normal) distribution
$\Phi^{ - 1}$: Inverse of the normal distribution
*θ*: Anisotropy angle of the correlogram
*v*: Separation distance between two points in the direction of the major axis along an elliptical contour
c_1_, c_2_, c_3_: Constants of the SSI equations
$C_{k}$: Covariance of cell k between two rainfields
*d*[*s*_*k*_]: Distance of a dry grid cell from the nearest wet grid cell
$\overline{d}$: An average of *d*[*s*_*k*_]
*F*^*−*^^*1*^: Inverse of distribution *F*
*F*_*C0*_: Zero-inflated fitted distributions of collocated dataset
*F*_*G0*_: Zero-inflated fitted distributions of rain gauge dataset
*F*_*S0*_: Zero-inflated fitted distribution of satellite precipitation product dataset
$F_{R}$: Two-parameter right-skewed distribution
*F*_*TRUE*_: “True” distribution
$F_{R0}$: Zero-inflated two-parameter right-skewed distribution of rainfall
*i*: Cell number within a moving window
*k*: A grid cell number
*L*_*u*_: Major axis length of the correlogram
*L*_*v*_: Minor axis length of the correlogram
*m*_*i*_: Weight of cell *i* within a moving window
*n*: Number of grid cells of SSI moving window
N: Number of grid cells of a rainfield
*p*: Probability
p_G0_: Corrected (true) rain gauge probability
*p*_*o*_: The ratio of the number of dry grid cells to the total number of grid cells
*p*_*s*_: Probability of SPP
Q: Range, difference between the maximum and minimum of values
*r*_*i*_: Rainfall amount of cell *i* within a moving window
$r\left[ {s_{k} } \right]$: Rainfall amount at location *S*_*k*_
*R*_*G*_: Rain gauge rainfall
*R(p)*: Rainfall amount given by the “true” distribution for a given p
*R*_*S0*_: Corrected (true) SPP rainfall
$R_{\Theta } \left( {u,v} \right)$: Two-dimensional (2D) exponential model in the elliptical contour coordinates
$R_{\Theta } \left( {x,y} \right)$: Two-dimensional (2D) exponential model in the cartesian coordinates
*R*_*O*_: Rainfield of maximum number of rain gauges
*R*_*P*_: Rainfield from reduced maximum number of rain gauges
*R*_*max*_: Maximum value of *R*_*O*_
*R*_*min*_: Minimum value of *R*_*O*_
*S*_*k*_: Coordinates of a grid cell
*u*: Separation distance between two points in the direction of the minor axis along an elliptical contour
U: Direction of the major axis of the correlogram
V: Direction of the minor axis of the correlogram
*w*: Gaussianised rainfields
*x*: Horizontal separation distance between two points in the cartesian coordinates
X: Cartesian coordinates in the horizontal direction
*y*: Vertical separation distance between two points in the cartesian coordinates
Y: Cartesian coordinates in the vertical direction

## References

[1] Gyasi-Agyei Y (2020). Identification of the optimum rain gauge network density for hydrological modelling based on radar rainfall analysis. Water.

[2] Maier R, Krebs G, Pichler M, Muschalla D, Gruber G (2020). Spatial rainfall variability in urban environments—high-density precipitation measurements on a city-scale. Water.

[3] Addi M, Gyasi-Agyei Y, Obuobie E, Amekudzi LK (2022). Evaluation of imputation techniques for infilling missing daily rainfall records on river basins in Ghana. Hydrol. Sci. J..

[4] Aguilera H, Guardiola-Albert C, Serrano-Hidalgo C (2020). Estimating extremely large amounts of missing precipitation data. J. Hydroinf..

[5] Kidd C, Becker A, Human GJ, Muller CL, Joe P, Skofronick-Jackson G, Kirschbaum DB (2017). So, how much of the Earth’s surface is covered by rain gauges?. Bull. Am. Meteorol. Soc..

[6] Adhikary SK, Muttil N, Yilmaz AG (2017). Cokriging for enhanced spatial interpolation of rainfall in two Australian catchments. Hydrol. Process..

[7] Hutchinson MF (1995). Interpolating mean rainfall using thin plate smoothing splines. Int. J. Geogr. Inf. Syst..

[8] Ly S, Charles C, Degre A (2011). Geostatistical interpolation of daily rainfall at catchment scale: The use of several variogram models in the Ourthe and Ambleve catchments, Belgium. Hydrol. Earth Syst. Sci..

[9] Chaplot V, Saleh A, Jaynes D (2005). Effect of the accuracy of spatial rainfall information on the modeling of water, sediment, and NO3–N loads at the watershed level. J. Hydrol..

[10] Xu H, Xu CY, Chen H, Zhang Z, Li L (2013). Assessing the influence of rain gauge density and distribution on hydrological model performance in a humid region of China. J. Hydrol..

[11] Villarini G, Krajewski WF (2010). Review of the different sources of uncertainty in single polarization radar-based estimates of rainfall. Surv. Geophys..

[12] Beck HE, van Dijk AIJM, Levizzani V, Schellekens J, Miralles DG, Martens B, de Roo A (2017). MSWEP: 3-hourly 0.25° global gridded precipitation (1979–2015) by merging gauge, satellite, and reanalysis data. Hydrol. Earth Syst. Sci..

[13] Li M, Shao Q (2010). An improved statistical approach to merge satellite rainfall estimates and raingauge data. J. Hydrol..

[14] Mitra AK, Bohra A, Rajeevan M, Krishnamurti T (2009). Daily Indian precipitation analysis formed from a merge of rain-gauge data with the TRMM TMPA satellite-derived rainfall estimates. J. Meteorol. Soc. Jpn. Ser..

[15] Hu Q, Li Z, Wang L, Huang Y, Wang Y, Li L (2019). Rainfall spatial estimations: A review from spatial interpolation to multi-source data merging. Water.

[16] Kidd CLV (2011). Status of satellite precipitation retrievals. Hydrol. Earth Syst. Sci..

[17] Novella NS, Thiaw WM (2013). African rainfall climatology version 2 for famine early warning systems. J. Appl. Meteorol. Climatol..

[18] Funk CC, Peterson PJ, Landsfeld MF, Pedreros DH, Verdin JP, Rowland JD, Romero BE, Husak GJ, Michaelsen JC, Verdin AP (2014). A quasi-global precipitation time series for drought monitoring. US Geol. Surv. Data Ser..

[19] Hersbach H (2020). The ERA5 global reanalysis. Q. J. R. Meteorol. Soc..

[20] Sun Q, Miao C, Duan Q, Ashouri H, Sorooshian S, Hsu KL (2018). A review of global precipitation data sets: Data sources, estimation, and inter-comparisons. Rev. Geophys..

[21] Islam MA, Yu B, Cartwright N (2020). Assessment and comparison of five satellite precipitation products in Australia. J. Hydrol..

[22] Zhang W, Brandt M, Guichard F, Tian Q, Fensholt R (2017). Using long-term daily satellite based rainfall data (1983–2015) to analyze spatio-temporal changes in the sahelian rainfall regime. J. Hydrol..

[23] Atiah WA, Amekudzi LK, Aryee JNA, Preko K, Danuor SK (2020). Validation of satellite and merged rainfall data over Ghana, West Africa. Atmosphere.

[24] Logah FY, Adjei KA, Obuobie E, Gyamfi C, Odai SN (2021). Evaluation and comparison of satellite rainfall products in the black volta basin. Environ. Process..

[25] Owusu C, Adjei KA, Odai SN (2019). Evaluation of satellite rainfall estimates in the Pra Basin of Ghana. Environ. Process..

[26] Amekudzi LK, Yamba EI, Preko K, Asare EO, Aryee J, Baidu M, Codjoe SNA (2015). Variabilities in rainfall onset, cessation and length of rainy season for the various agro-ecological zones of Ghana. Climate.

[27] WRC. *Pra River Basin—Integrated Water Resources Management Plan* (Water Resources Commission, Accra, 2012).

[28] Gyasi-Agyei Y (2022). A framework for comparing two rainfields based on spatial structure: A case of radar against selected satellite precipitation products over southeast Queensland, Australia. J. Hydrol..

[29] Gyasi-Agyei Y, Pegram G (2014). Interpolation of daily rainfall networks using simulated radar fields for realistic hydrological modelling of spatial rain field ensembles. J. Hydrol..

[30] Gyasi-Agyei Y (2016). Assessment of radar based locally varying anisotropy on daily rainfall interpolation. Hydrol. Sci. J..

[31] Gyasi-Agyei Y (2018). Realistic sampling of anisotropic correlogram parameters for conditional simulation of daily rainfields. J. Hydrol..

[32] Gyasi-Agyei Y (2019). Propagation of uncertainties in interpolated rainfields to runoff errors. Hydrol. Sci. J..

[33] Jones EL, Rendell L, Pirotta E, Long JA (2016). Novel application of a quantitative spatial comparison tool to species distribution data. Ecol. Ind..

[34] Wang Z, Bovik AC, Sheikh HR, Member S, Simoncelli EP, Member S (2004). Image quality assessment: From error visibility to structural similarity. IEEETrans. Image Process..

[35] Gudmundsson L, Bremnes JB, Haugen JE, Engen-Skaugen T (2012). Downscaling RCM precipitation to the station scale using statistical transformations-a comparison of methods. Hydrol. Earth Syst. Sci..

[36] Velasco-Forero CA, Sempere-Torres D, Cassiraga EF, Gómez-Hernández JJ (2009). A non-parametric automatic blending methodology to estimate rainfall fields from rain gauge and radar data. Adv. Water Resour..

[37] Sinclair S, Pegram GGS (2005). Combining radar and rain gauge rainfall estimates using conditional merging. Atmos. Sci. Lett..

[38] Arkin PA, Meisner BN (1987). The relationship between large scale convective rainfall and cold cloud over the western hemisphere during 1982-1984. Mon. Weather Rev..

[39] Derin A, Yilmaz KK (2014). Evaluation of multiple satellite-based precipitation products over complex topography. J. Hydrometeorol..

[40] Prakash S (2019). Performance assessment of CHIRPS, MSWEP, SM2RAIN-CCI, and TMPA precipitation products across India. J. Hydrol..

[41] Gyasi-Agyei Y, de Troch FP, Troch PA (1996). A dynamic hillslope response model in a geomorphology based rainfall-runoff model. J. Hydrol..

