# Supplementary material for: Optimal selection of daily satellite precipitation product based on structural similarity index at 1 km resolution for the Pra catchment, Ghana
Source: Sci Rep. 2023 Oct 4;13:16702. doi: 10.1038/s41598-023-43075-0 (PMC10550986; doi:10.1038/s41598-023-43075-0)
Supplement: Supplementary file 1 — Supplementary Information. [file 41598_2023_43075_MOESM1_ESM.pdf]

Supplementary Material:

Optimal selection of daily satellite precipitation product based on structural similarity index at 1 km resolution for the Pra catchment, Ghana

Yeboah Gyasi-Agyei[1]*, Emmanuel Obuobie[2], Bofu Yu[1], Martin Addi[3,4], Bashiru Yahaya[5]

[1]School of Engineering and Built Environment, Griffith University, Nathan, Australia
[2]Water Research Institute, Council for Scientific and Industrial Research, Accra, Ghana
[3]Meteorology and Climate Science Department, Kwame Nkrumah University of Science and Technology, Kumasi, Ghana
[4]Remote Sensing and Climate Centre, Ghana Space Science and Technology Institute, Ghana Atomic Energy Commission, Accra, Ghana
[5]Ghana Meteorological Agency, Accra, Ghana
***Corresponding author (y.gyasi-agyei@griffith.edu.au)**

1 A brief description of the satellite precipitation products (SPPs)

ARC2 (Africa Rainfall Climatology Version 2) merges sparse daily rain gauge data over Africa from Global Telecommunication System (GTS) maintained by the Climate Prediction Center (CPC) and 3-hourly geostationary infrared (IR) operated by the European Organization for the Exploitation of Meteorological Satellites[1] (EUMETSAT) (https://iridl.ldeo.columbia.edu/SOURCES/.NOAA/.NCEP/.CPC/.FEWS/.Africa/.DAILY/.ARC2/.daily/index.html).

CHIRPS (Climate Hazards Group InfraRed Precipitation with Station data) merges observed precipitation (e.g., GTS, GHCN (Global Historical Climatology Network), GSOD (Global Summary of the Day) with IR satellite data from different sources such as NOAA and the CPC[2] (https://www.chc.ucsb.edu/data/chirps).

CMORPH (CPC MORPHing technique) uses geostationary satellite IR brightness temperature extracted from several satellites and precipitation derived from PMW sensors of polar-orbiting satellites[3] (https://www.cpc.ncep.noaa.gov/products/janowiak/cmorph_description.html).

ERA5 (European ReAnalysis) is the fifth generation ECMWF (European Centre for Medium-Range Weather Forecasts) atmospheric reanalysis product that combines historical observations (satellite and ground radar precipitation composites) from several sources into global estimates using advanced modelling and data assimilation systems[4] (https://www.ecmwf.int/en/forecasts/dataset/ecmwf-reanalysis-v5).

GSMAP (Global Satellite Mapping of Precipitation) uses multi-band PMW and IR radiometers from multiple satellites including the GPM Core Observatory satellite. NOAA/CPC gauge measurements are used to adjust the precipitation estimates[5] (https://sharaku.eorc.jaxa.jp/GSMaP/).

IMERG (Integrated Multi-satellitE Retrievals for GPM) uses PMW and IR sensors data from the satellite constellation operated by GPM. The estimated precipitations are adjusted using the monthly Global Precipitation Climatology Project (GPCP) gauge data[6,7] (https://disc.gsfc.nasa.gov/datasets/GPM_3IMERGHH_06/summary).

MSWEP (Multi-Source Weighted-Ensemble Precipitation) precipitation product hosted by Princeton Climate Analytics merges satellite, gauge and reanalysis data. The gauge data sources include GHCN-D (GHCN-Daily), GPCC (Global Precipitation Climatology Centre) and GSOD. The satellite product sources include GSMAP, CMORPH and TMPA 3B42RT, and the reanalyses data sources include the ECMWF, ERA-Interim and JRA-55 (Japanese 55-year reanalysis)[8] (http://www.gloh2o.org/mswep/).

Three PERSIANN (Precipitation Estimation from Remotely Sensed Information using Artificial Neural Networks) SPPs, hosted by the Center for Hydrometeorology and Remote Sensing at the University of California, Irvine, were evaluated (PERSIANN, PERSIANN-CCS and PERSIANN-DIR). The PERSIANN products uses IR and visible imagery from global geosynchronous satellites to estimate rainfall. PERSIANN-CCS (PERSIANN-Cloud Classification System, PERCCS) is a real-time high-resolution version that uses a variable

threshold cloud segmentation algorithm to identify individual cloud patches. It relies on TMPA and ground radar observations for the initial training of the intensity and distribution of rainfall. PERSIANN-DIR (PERSIANN dynamic infrared rain, PERDIR) is also a high resolution version and relies on high frequency sampled IR imagery for shortening the time of occurrence of rainfall[9] (https://chrsdata.eng.uci.edu/).

TAMSAT (Tropical Applications of Meteorology using SATellite and ground based observations) uses Meteosat thermal IR imagery from EUMETSAT to estimate rainfall and calibrated by rain gauge observations. The thermal IR imagery monitors the cold cloud tops of convective rainfall and is more suitable for the tropics but has limitations where warm rain dominates and also over mountain ranges[10] (http://www.tamsat.org.uk/sites/data-download/index.html).

TMPA 3B42RT (Tropical Rainfall Measuring Mission Multi-Satellite Precipitation Analysis real time) combines PMW and IR sensors and retrieval algorithms, and its calibrated using monthly rain gauge data. It was a joint mission between NASA and the Japan Aerospace Exploration (JAXA) and ended on 31/12/2019[11]. https://disc2.gesdisc.eosdis.nasa.gov/data/TRMM_RT/TRMM_3B42RT.7/).

W5E5V2 is a merger of WFDE5 dataset over land and ERA5 dataset over the ocean (https://doi.org/10.48364/ISIMIP.342217). It was developed to support bias correction of input data for the phase 3b of the Inter-Sectoral Impact Model Intercomparison Project. The WFDE5 dataset was based on the WATCH (WATer and global CHange) Forcing Data (WFD) methodology developed for meteorological variables in ERA5[12].

2 Examples of the developed 1 km x 1 km daily rainfields

SSI indices values shown on the conditionally merged fields are those of the prior and post bias correction scenarios of the SPPs compared with the rain gauge field considered as the ground truth.

Day 2017-03-06 has the lowest number of operational gauges (20) (Table S1) and was one of the days that the gauges were wetter than the SPPs. PERCSS was the best SPP for Scenario 1 (prior to bias correction) with the SSIM value of 0.597 (Table S2) but failed to capture the three highest rain gauge readings in the central part of the catchment (Figure S1). In the case of Scenario 2 (after bias correction), CHIRPS emerged as the best SPP. From Figure S1a, the rain gauge wetness was higher than that of CHIRPS, and also recorded higher rainfall amounts resulting in its CDF being below that of CHIRPS. Also, the collocated CDF was close to that of the full CHIRPS, meaning the rain gauge CDF is also similar to the bias corrected CDF. This resulted in no change in SSIM value (0.56) post bias correction as the improvement in SIM was offset by the decline in SIV, SIP remaining largely unchanged (Figures S1c and S1d; Table S2). The correlograms of both PERCSS and CHIRPS are similar except for the anisotropic directions that are 24.8° and 123.5°, respectively (Table S3). Despite this day having had only 20 fairly distributed operational rain gauges with 55% being wet over the catchment, the merged results are quite reasonable (Figures S1g and S1h) but performance of the prior bias corrected PERCCS was preferred because it has a higher SSIM value (Table S2).

One of the days that the SPPs performed poorly was 2017-04-25, with the best SPP of Scenario 1 of TMPA scoring 0.311 for SSIM (Table S2). It missed or underestimated the rain gauge readings (Figure S2b). For Scenario 2, the best SPP was PERDIR but with the bias correction decreasing the SSIM value from 0.219 to 0.198 (Table S2). The CDF patterns shown in Figure S2a are like those observed for 2017-03-06, though for different SPPs. The correlograms for both TMPA and PERDIR are similar with a pronounced anisotropy, and the

anisotropic directions being 22.5° and 35.8°, respectively (Table S3). Having a higher SSIM value, the merged rainfield of TMPA was preferred (Table S2).

The best SPPs of Scenario 1 (PERDIR) and Scenario 2 (ARC2) both indicate heavy rainfall activities in the northwest region, and also a narrow middle belt for PERDIR, but unfortunately there are no operational rain gauges in these areas for 2017-08-03 (Figures S3b and S3c). While the rain gauges are indicating 100% wetness for this day, the best SPPs show dryness in the southern part of the catchment, and also the eastern part by ARC2. It is interesting to note the CDF of the collocated ARC2, resulting from very a few wet rain gauges recording low rainfall amounts, is above the CDF of ARC2. Thus, the CDF of the rain gauges had to be shifted to the right to obtain the bias corrected CDF (Figure S3a). The bias correction improved ARC2 with SSIM increasing from 0.072 to 0.397, being slightly higher than the value yielded by PERDIR (0.347) and rendering it 100% wetness (Table S2). The correlogram of ARC2 has longer correlation lengths and a more pronounced anisotropy (Table S3, Figures S3e and S3f) and so do the merged rainfields (Figures S3g and S3h), but the ARC2 rainfield was preferred because it has a higher SSIM value (Table S2).

The first example of a day having over 60 operational rain gauges is 2020-06-18 which had rainfall concentrated on the northwest section of the catchment. IMERG was the best SPP for Scenario 1 with an SSIM value of 0.299 weighed down by the low value of 0.571 for SIP (Table S2). This SPP tracked the direction of the storm quite well (Figure S4b). This was also the case for PERDIR which emerged as the best SPP for Scenario 2 (Figure S4c) but generally has 100% wetness compared with that of the rain gauges (Figure S4a). However, after bias correcting, PERDIR of the dry areas become more pronounced (Figure S4d) with an improvement in SIM, SIV, SIP and SSIM by 0.048, 0.105, 0.083 and 0.126, respectively, over the Scenario 1 best SPP of IMERG (Table S2). Also of note is that the bias corrected CDF is the same as that of the rain gauges because of the similarity of the CDFs of the SPP and the

collocated one (Figure S4a). For both scenarios 1 and 2, the best SPP yielded similar correlograms with strong anisotropy (η=0.6) in the northeast direction (Table S3, Figures S4e and S4f). The merged rainfield of Scenario 2 (PERDIR) was preferred because it has a higher SSIM value (0.424 compared with 0.299).

Day 2022-06-22 is the second example with over 60 operational rain gauges and its results are depicted in Figure S5. For this day PERDIR was the best Scenario 1 SPP that recorded SSIM value of 0.342 (Figure S5b). For Scenario 2, ERA5 emerged as the best SPP (Figure S5d) with SSIM value of 0.633 (over 85% increase) contributed by the significant increases in SIM (41.1%), SIV (0.07%) and SIP (25%) compared with Scenario 1 best SPP values. As in many cases, ERA5 exhibits 100% wetness compared with the rain gauges (50% wetness) as demonstrated in Figure S5ac, but the bias correction improved ERA5 where the dry areas became more pronounced (Figure S5d). The Scenario 1 (PERDIR) correlogram does not exhibit anisotropy (η=0.88) and has shorter axis lengths compared with the Scenario 2 best SPP (ERA5, mild anisotropy η=0.7) (Table S3). This is reflected in the merged rainfields shown in Figures S5g and S5h, preferring that of Scenario 2 (ERA5) because it has the higher SSIM value of 0.633 (Table S2).

MSWEP was the best SPP for Scenario 1 having SSIM value of 0.623 for 2020-07-04. From Figure S6b, it captured the rain gauge rainfall pattern very well (SIP=0.95), but it recorded between 0.1 and 1 mm for the significantly large dry areas indicating 100% wetness against the rain gauge value of 0.62. Although the best Scenario 2 SPP (PERDIR) indicated dry areas, it missed the dry areas in the upper part of the catchment (Figure S6c). However, after the bias correction most dry areas were exposed with the SSIM increasing from 0.545 to 0.604 (Figure S6c) but fell slightly below that of scenario 1 (0.623). The CDF of PERDIR (was slightly below the collocated counterpart causing a slight shift of the CDF of the rain gauge to the right to achieve the bias corrected CDF (Figure S6a). Strong anisotropy (η=0.6) in the

northwest direction is observed in the correlogram of MSWEP which also has longer axis lengths compared with those of PERDIR (η=0.7) for this day (Figures S6c and S6d), which is reflected in the merged rainfields (Figures S6g and S6h). Although Scenario 1 is preferred, Scenario 2 results gave a very high value of SSIM.

The best Scenario 1 SPP for 2020-11-25 was GSMAP (SSIM=0.349), despite it having missed some dry areas and also having indicated dry areas in some wet sections (Figure S7b). ERA5 was selected as the best SPP for Scenario 2 and demonstrates 100% wetness against the rain gauge value of 0.49 (Figure S7c, Table S1). However, the bias correction improved the rainfield with SSIM increasing from 0.187 to 0.348 (Figure S7d) and also showing some of the dry areas. While the lower part of the CDF of the collocated data matched that of the full ERA5 rainfield, the upper part was higher and thus called for a minimal shift of the rain gauge CDF to the right to obtain the bias corrected CDF. Both GSMAP and ERA5 display similar correlograms except with a mild anisotropy of that of ERA5 (Table S3). From Figures S7g and S7h, the merged rainfields are similar, but ERA5 is giving very low rainfall amounts (<1 mm) for some of the dry areas exhibited by GSMAP.

Stronger anisotropy observed for most rainy days should encourage the use of anisotropic correlograms for rainfall modelling for the Pra catchment. The anisotropy could be related to the relief and wind direction.

References

1 Novella, N. S., & Thiaw, W. M. African Rainfall Climatology Version 2 for Famine Early Warning Systems. *J. Appl. Meteorol. Climatol.* **52**, 588–606, doi:https://doi.org/10.1175/JAMC-D-11-0238.1 (2013).

2 Funk, C. C., Peterson, P. J., Landsfeld, M. F., Pedreros, D. H., Verdin, J. P., Rowland, J. D., Romero, B. E., Husak, G. J., Michaelsen, J. C. & Verdin, A. P. A quasi-global precipitation time series for drought monitoring:. 4 (2014).

3 Joyce, R. J., et al. . CMORPH: A method that produces global precipitation estimates from passive microwave and infrared data at high spatial and temporal resolution. *J.*

Hydrometeorol. **5**, 487–503, doi:https://doi.org/10.1175/1525-7541(2004)005%3C0487:CAMTPG%3E2.0.CO;2 (2004).

4 Hersbach, H., et al. The ERA5 global reanalysis. *Q J R Meteorol Soc.,* **146**, 1999–2049, doi:https://doi.org/10.1002/qj.3803 (2020).

5 Ushio, T., et al. A Kalman Filter Approach to the Global Satellite Mapping of Precipitation (GSMaP) from Combined Passive Microwave and Infrared Radiometric Data *J. Meteorol. Soc. Japan A.* **87**, 137-151, doi:https://doi.org/10.2151/jmsj.87A.137 (2009).

6 Hou A.Y., et al. The global precipitation measurement mission. *Bull. Amer. Meteor. Soc.* **95**, 701–722, doi:https://doi.org/10.1175/BAMS-D-13-00164.1 (2014).

7 Huffman, G. J., Stocker, E. F., Bolvin, D .T., Nelkin, E. J. & Tan, J. (ed MD Greenbelt, Goddard Earth Sciences Data and Information Services Center (GES DISC).) (2019).

8 Beck, H. E., Wood, E. F., Pan, M., Fisher, C. K., Miralles, D. M., van Dijk, A. I. .J. M., McVicar, T. R. & Adler, R. F. MSWEP V2 global 3-hourly 0.1° precipitation: methodology and quantitative assessment. *Bull. Amer. Meteor. Soc.* **100**, 473–500, doi:https://doi.org/10.1175/BAMS-D-17-0138.1 (2019).

9 Sorooshian, S., Hsu, K., Gao, X., Gupta, H. V., Imam, B. & Braithwaite D. Evaluation of PERSIANN system satellite-based estimates of tropical rainfall. *Bull. Am. Meteorol. Soc.* **81**, 2035–2046, doi:https://doi.org/10.1175/1520-0477(2000)081%3C2035:EOPSSE%3E2.3.CO;2 (2000).

10 Maidment, R. I., et at. A new, long-term daily satellite-based rainfall dataset for operational monitoring in Africa. *Sci. Data* **4**, 170063, doi:https://doi.org/10.1038/sdata.2017.63 (2017).

11 Huffman, G. J., Adler, R. F., Bolvin, D. T. & Nelkin, E. J.,. in *Satellite Rainfall Applications for Surface Hydrology* (ed M. Gebremichael, Hossain, F.) (Springer, Dordrecht, 2010).

12 Cucchi, M., Weedon, G. P., Amici, A., Bellouin, N., Lange, S., Müller Schmied, H., Hersbach, H. & Buontempo, C. WFDE5: bias-adjusted ERA5 reanalysis data for impact studies. *Earth Syst. Sci. Data* **12**, 2097-2120, doi:https://doi.org/10.5194/essd-12-2097-2020 (2020).

Table S1. Properties of the rain gauge data within the 20 km buffer of the selected dates.

Date	NG	NW	MAX (mm)	MEAN (mm)	WetPro
2017-03-06	20	11	33.3	10.3	0.550
2017-04-25	27	18	57.8	19.6	0.667
2017-08-03	24	23	15.9	3.9	0.958
2020-06-18	64	51	113.2	26.7	0.797
2020-06-22	66	33	39.2	7.4	0.500
2020-07-04	68	42	114.4	11.2	0.618
2020-11-25	63	31	36.9	13.1	0.492

NG – total number of gauges; NW– number of wet gauges; MAX – maximum wet gauge amount; MEAN – mean of wet gauges; WP – proportion of wet gauges

Table S2. Best Satellite Precipitation Product (SPP) for the selected days and their structural similarity indices values of window size of 11km x 11km considered for the cases prior (Scenario 1) and post bias correction (Scenario 2).

Date	Scenario 1 (Prior Bias Correction)					Scenario 2 (Post Bias Correction)				
	BEST SPP	SSIM	SIM	SIV	SIP	BEST SPP	SSIM	SIM	SIV	SIP
2017-03-06	PERCCS	0.597	0.605	0.986	0.998	CHIRPS	0.560	0.633	0.879	0.941
2017-04-25	TMPA	0.311	0.350	0.743	0.861	PERDIR	0.198	0.454	0.662	0.684
2017-08-03	PERDIR	0.347	0.711	0.883	0.547	ARC2	0.397	0.708	0.859	0.687
2020-06-18	IMERG	0.299	0.652	0.779	0.571	PERDIR	0.425	0.700	0.884	0.654
2020-06-22	PERDIR	0.342	0.484	0.793	0.737	ERA5	0.633	0.683	0.851	0.921
2020-07-04	MSWEP	0.623	0.713	0.852	0.950	PERDIR	0.604	0.692	0.929	0.869
2020-11-25	GSMAP	0.349	0.430	0.626	0.879	ERA5	0.348	0.460	0.633	0.840

SIM - mean similarity index, SIV - variance similarity index, SIP - spatial pattern similarity index, SSIM - overall measure of similarity

Table S3. Correlogram parameters for the best SPP for the selected dates for the cases prior (S1) and post (S2) bias correction.

Date	Scenario 1 (Prior Bias Correction)					Scenario 2 (Post Bias Correction)				
	BEST SPP	L_u (km)	L_v (km)	η	θ (°)	BEST SPP	L_u (km)	L_v (km)	η	θ (°)
2017-03-06	PERCCS	54.3	43.4	0.80	24.8	CHIRPS	50.0	37.8	0.76	123.5
2017-04-25	TMPA	50.5	34.0	0.67	22.5	PERDIR	50.5	31.8	0.63	35.8
2017-08-03	PERDIR	62.0	38.7	0.62	26.4	ARC2	90.2	48.2	0.53	48.5
2020-06-18	IMERG	86.7	49.9	0.58	34.7	PERDIR	86.1	51.6	0.60	39.5
2020-06-22	PERDIR	32.4	28.4	0.88	1.8	ERA5	48.4	33.9	0.70	157.1
2020-07-04	MSWEP	72.5	41.6	0.57	121.1	PERDIR	28.6	20.1	0.70	7.5
2020-11-25	GSMAP	71.2	54.8	0.77	6.5	ERA5	65.1	47.2	0.72	22.1

L_v - minor axis length, L_u - minor axis length, η - anisotropy ratio, θ - anisotropy angle measured anticlockwise from the x-axis.

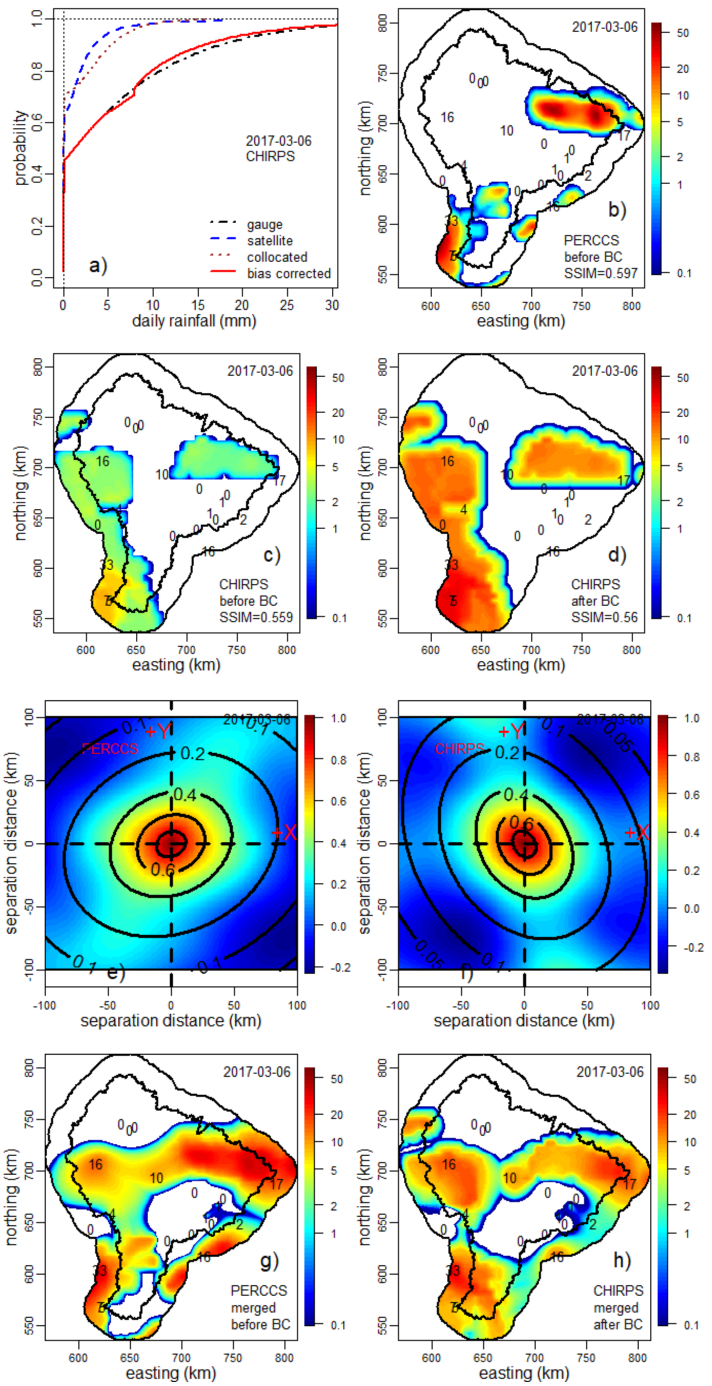

Figure S1: Processes for generating the 1 km x 1 km rainfield (mm) for 2017-03-06: a) determination of the true probability distribution function, b) PERCCS - the best satellite precipitation product (SPP) before bias correction, c) CHIRPS - before bias correction, d) CHIRPS – the best SPP after bias correction, e) correlogram derived from PERCCS, f) correlogram derived from CHIRPS, g) conditional merging of gauge and PERCCS (Scenario 1), h) conditional merging of gauge and bias corrected CHIRPS (Scenario 2). Rain gauge readings are shown on the rainfields noting that they represent only one grid cell each so are exaggerated on the maps. Indicated SSIM values are for the largest window size of 11 km x 11 km. All maps were produced using R software version 4.2.1 (https://cran.r-project.org/).

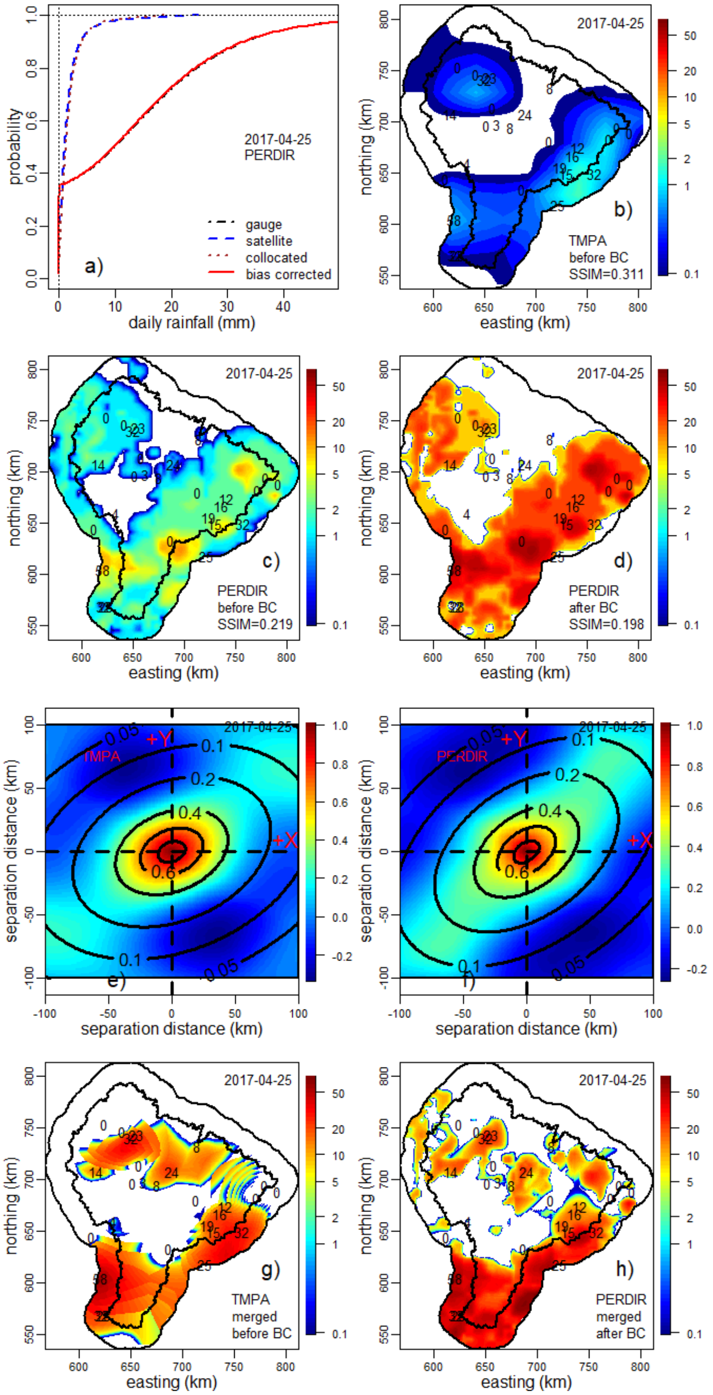

Figure S2: Processes for generating the 1 km x 1 km rainfield (mm) for 2017-04-25: a) determination of the true probability distribution function, b) ERA5 - the best satellite precipitation product (SPP) before bias correction, c) PERDIR - before bias correction, d) PERDIR – the best SPP after bias correction, e) correlogram derived from ERA5, f) correlogram derived from TAMSAT, g) conditional merging of gauge and ERA5 (Scenario 1), h) conditional merging of gauge and bias corrected PERDIR (Scenario 2). Rain gauge readings are shown on the rainfields noting that they represent only one grid cell each so are exaggerated on the maps. Indicated SSIM values are for the largest window size of 11 km x 11 km. All maps were produced using R software version 4.2.1 (https://cran.r-project.org/).

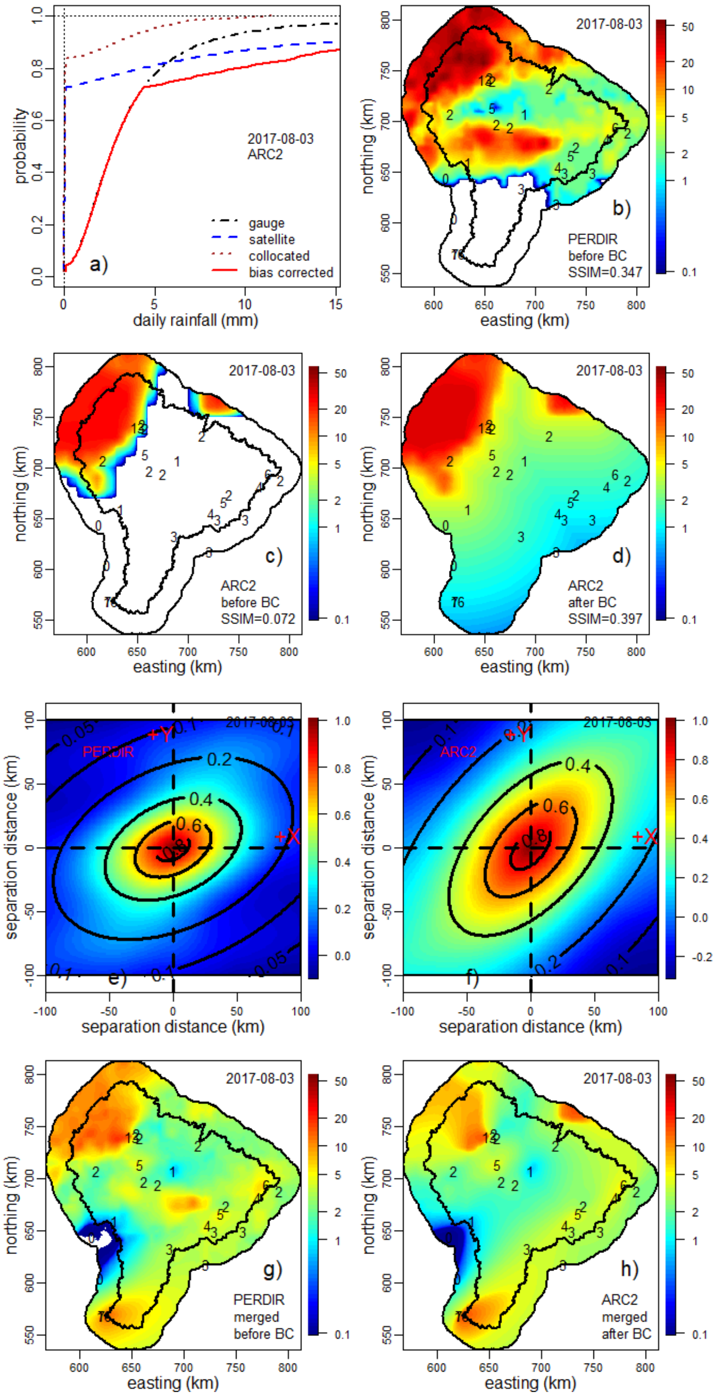

Figure S3: Processes for generating the 1 km x 1 km rainfield (mm) for 2017-08-03: a) determination of the true probability distribution function, b) PERDIR - the best satellite precipitation product (SPP) before bias correction, c) PERCCS - before bias correction, d) PERCCS – the best SPP after bias correction, e) correlogram derived from PERDIR, f) correlogram derived from PERCCS, g) conditional merging of gauge and PERDIR (Scenario 1), h) conditional merging of gauge and bias corrected PERCCS (Scenario 2). Rain gauge readings are shown on the rainfields noting that they represent only one grid cell each so are exaggerated on the maps. Indicated SSIM values are for the largest window size of 11 km x 11 km. All maps were produced using R software version 4.2.1 (https://cran.r-project.org/).

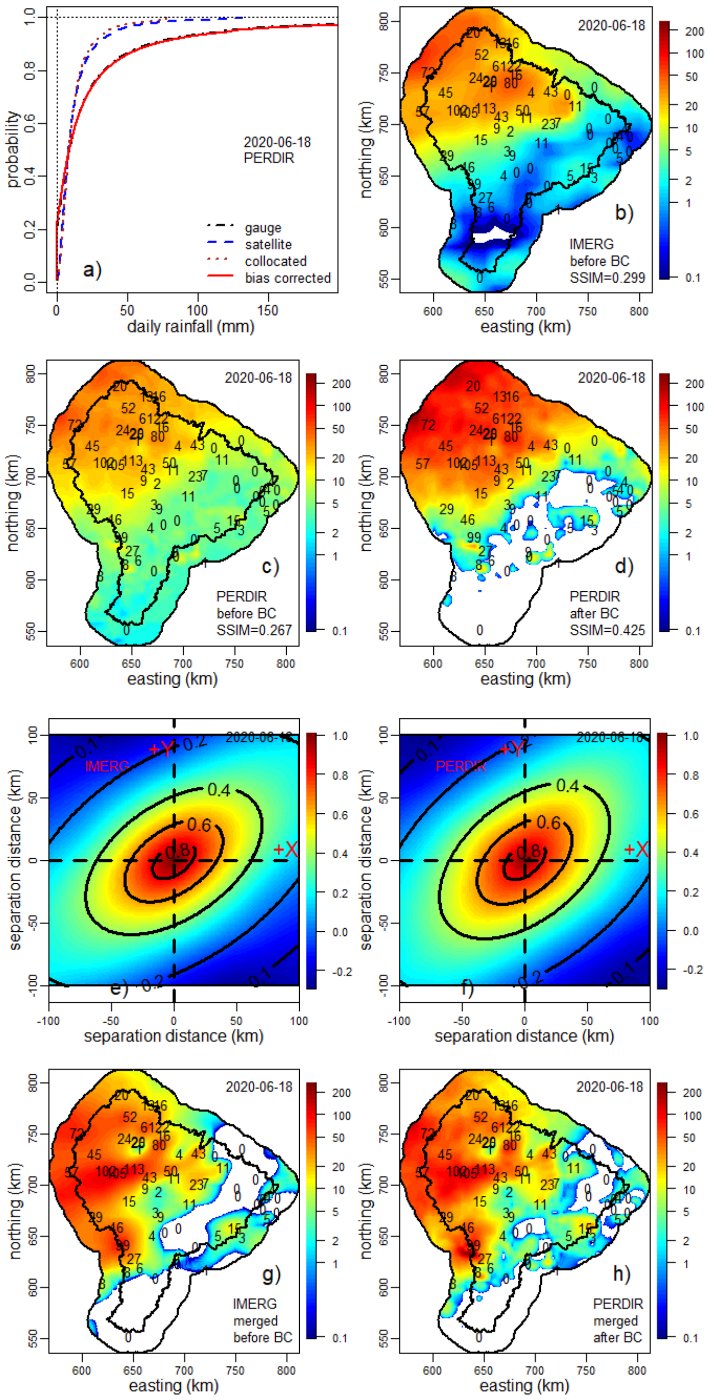

Figure S4: Processes for generating the 1 km x 1 km rainfield (mm) for 2020-06-18: a) determination of the true probability distribution function, b) IMERG - the best satellite precipitation product (SPP) before bias correction, c) PERDIR - before bias correction, d) PERDIR – the best SPP after bias correction, e) correlogram derived from IMERG, f) correlogram derived from PERDIR, g) conditional merging of gauge and IMERG (Scenario 1), h) conditional merging of gauge and bias corrected PERDIR (Scenario 2). Rain gauge readings are shown on the rainfields noting that they represent only one grid cell each so are exaggerated on the maps. Indicated SSIM values are for the largest window size of 11 km x 11 km. All maps were produced using R software version 4.2.1 (https://cran.r-project.org/).

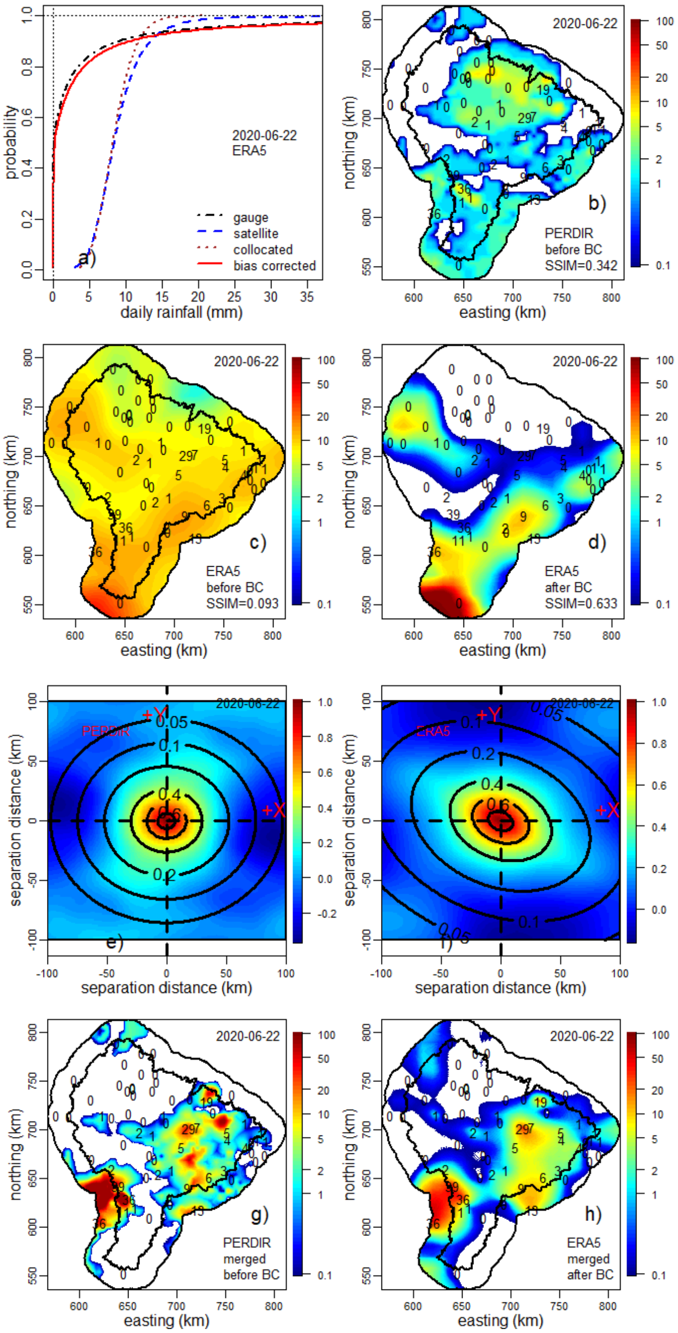

Figure S5: Processes for generating the 1 km x 1 km rainfield (mm) for 2020-06-22: a) determination of the true probability distribution function, b) PERDIR - the best satellite precipitation product (SPP) before bias correction, c) ERA5 - before bias correction, d) ERA5 – the best SPP after bias correction, e) correlogram derived from PERDIR before bias correction, f) correlogram derived from the bias corrected ERA5, g) conditional merging of gauge and PERDIR (Scenario 1), h) conditional merging of gauge and bias corrected ERA5 (Scenario 2). Rain gauge readings are shown on the rainfields noting that they represent only one grid cell each so are exaggerated on the maps. Indicated SSIM values are for the largest window size of 11 km x11 km. All maps were produced using R software version 4.2.1 (https://cran.r-project.org/).

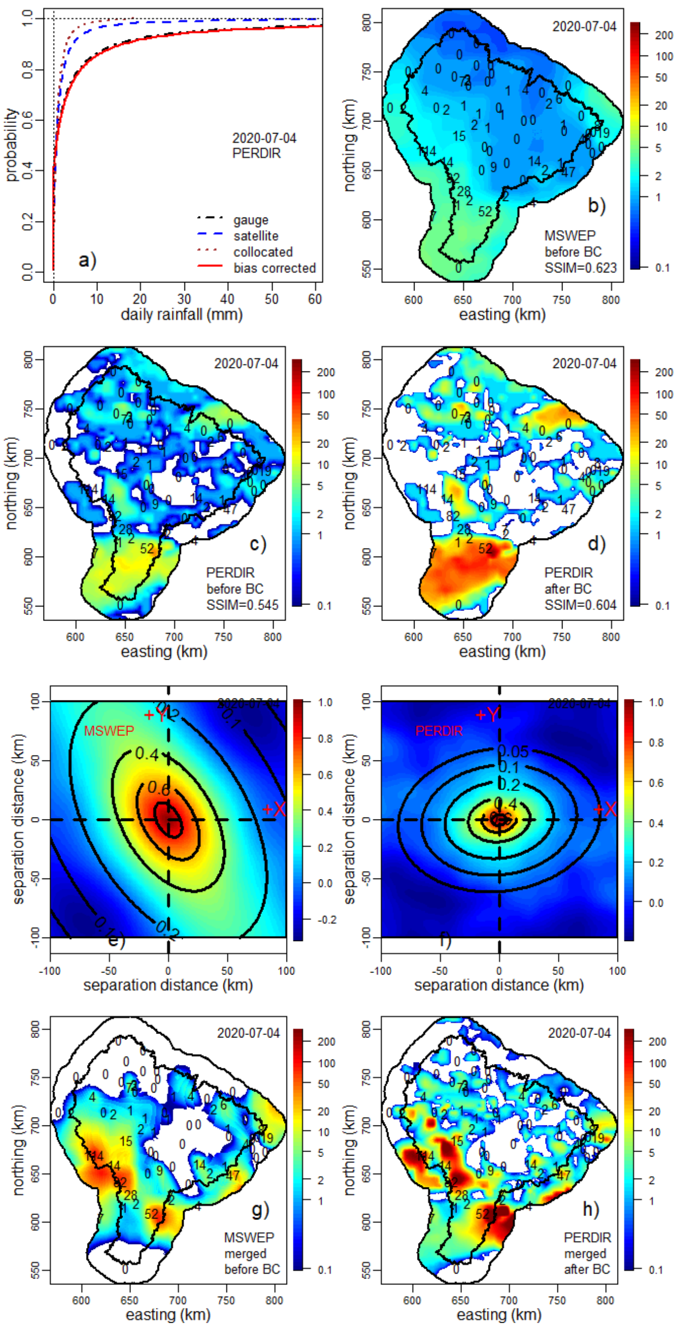

Figure S6: Processes for generating the 1 km x 1 km rainfield (mm) for 2020-07-04: a) determination of the true probability distribution function, b) MSWEP - the best satellite precipitation product (SPP) before bias correction, c) PERDIR - before bias correction, d) PERDIR – the best SPP after bias correction, e) correlogram derived from MSWEP, f) correlogram derived from PERDIR, g) conditional merging of gauge and MSWEP (Scenario 1), h) conditional merging of gauge and bias corrected PERDIR (Scenario 2). Rain gauge readings are shown on the rainfields noting that they represent only one grid cell each so are exaggerated on the maps. Indicated SSIM values are for the largest window size of 11 km x 11km. All maps were produced using R software version 4.2.1 (https://cran.r-project.org/).

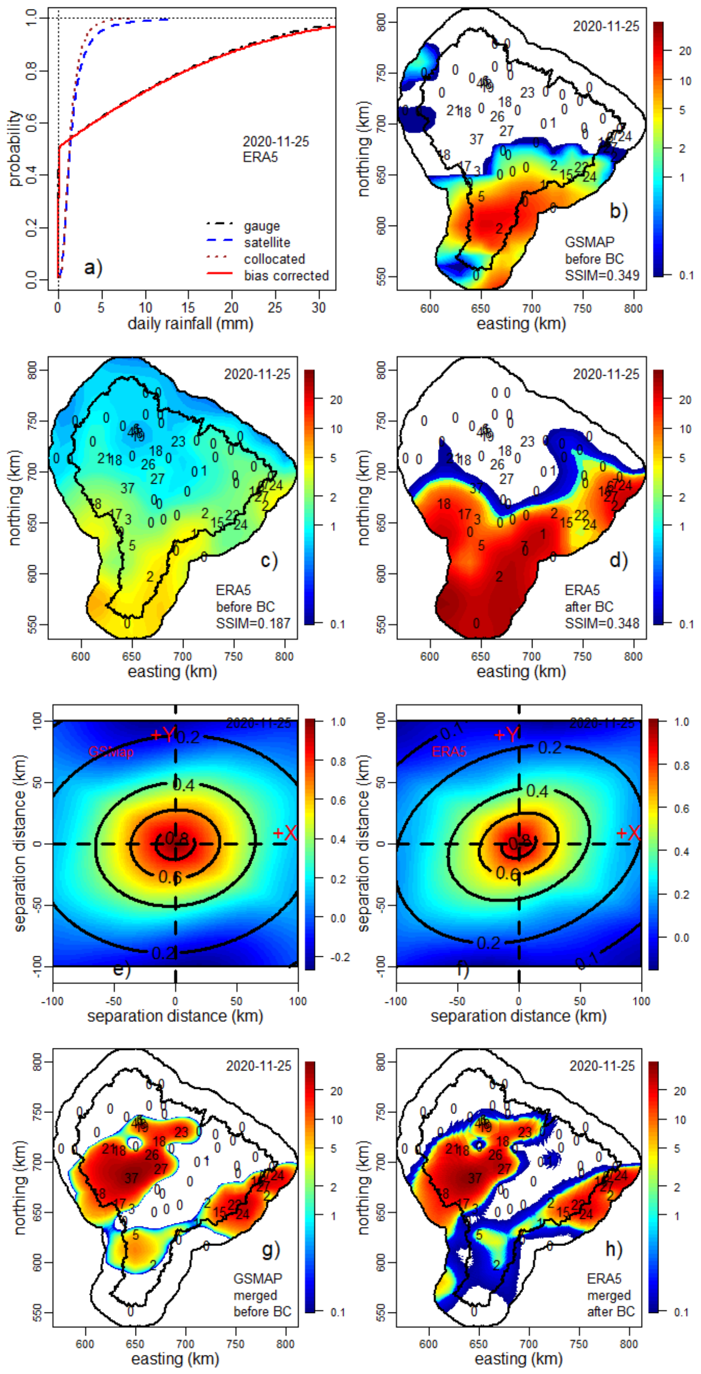

Figure S7: Processes for generating the 1 km x 1 km rainfield (mm) for 2020-11-25: a) determination of the true probability distribution function, b) GSMAP - the best satellite precipitation product (SPP) before bias correction, c) ERA5- before bias correction, d) ERA5– the best SPP after bias correction, e) correlogram derived from GSMAP, f) correlogram derived from ERA5, g) conditional merging of gauge and GSMAP (Scenario 1), h) conditional merging of gauge and bias corrected ERA5 (Scenario 2). Rain gauge readings are shown on the rainfields noting that they represent only one grid cell each so are exaggerated on the maps. Indicated SSIM values are for the largest window size of 11 km x 11 km. All maps were produced using R software version 4.2.1 (https://cran.r-project.org/).
